# Enhancing adoptive T‐cell therapy with fucoidan‐based IL‐2 delivery microcapsules

**DOI:** 10.1002/btm2.10362

**Published:** 2022-07-05

**Authors:** Eun Young Jeon, Da‐som Choi, Seunghyun Choi, Ju‐young Won, Yunju Jo, Hye‐bin Kim, Youngmee Jung, Sang Chul Shin, Hophil Min, Hae Woong Choi, Myeong Sup Lee, Yoon Park, Justin J. Chung, Hyung‐seung Jin

**Affiliations:** ^1^ Center for Biomaterials Biomedical Research Institute, Korea Institute of Science and Technology (KIST) Seoul South Korea; ^2^ Department of Convergence Medicine, Asan Institute for Life Sciences, Asan Medical Center University of Ulsan College of Medicine Seoul South Korea; ^3^ Theragnosis Center Biomedical Research Institute, Korea Institute of Science and Technology (KIST) Seoul South Korea; ^4^ Department of Life Sciences Korea University Seoul South Korea; ^5^ School of Electrical and Electronic Engineering Yonsei University Seoul South Korea; ^6^ Yonsei‐KIST Convergence Research Institute Seoul South Korea; ^7^ Technology Support Center Korea Institute of Science and Technology (KIST) Seoul South Korea; ^8^ Doping Control Center Korea Institute of Science and Technology (KIST) Seoul South Korea; ^9^ Department of Biomedical Sciences University of Ulsan College of Medicine Seoul South Korea; ^10^ Transdisciplinary Department of Medicine and Advanced Technology Seoul National University Hospital Seoul South Korea; ^11^ Department of Medicine Seoul National University College of Medicine Seoul South Korea

**Keywords:** adoptive T‐cell therapy, complex coacervate, fucoidan, immunotherapy, interleukin‐2

## Abstract

Adoptive cell therapy (ACT) with antigen‐specific T cells is a promising treatment approach for solid cancers. Interleukin‐2 (IL‐2) has been utilized in boosting the efficacy of ACT. However, the clinical applications of IL‐2 in combination with ACT is greatly limited by short exposure and high toxicities. Herein, a complex coacervate was designed to intratumorally deliver IL‐2 in a sustained manner and protect against proteolysis. The complex coacervate consisted of fucoidan, a specific IL‐2 binding glycosaminoglycan, and poly‐*l*‐lysine, a cationic counterpart (FPC^2^). IL‐2‐laden FPC^2^ exhibited a preferential bioactivity in ex vivo expansion of CD8^+^T cells over Treg cells. Additionally, FPC^2^ was embedded in pH modulating injectable gel (FPC^2^‐IG) to endure the acidic tumor microenvironment. A single intratumoral administration of FPC^2^‐IG‐IL‐2 increased expansion of tumor‐infiltrating cytotoxic lymphocytes and reduced frequencies of myeloid populations. Notably, the activation and persistency of tumor‐reactive T cells were observed only in the tumor site, not in the spleen, confirming a localized effect of FPC^2^‐IG‐IL‐2. The immune‐favorable tumor microenvironment induced by FPC^2^‐IG‐IL‐2 enabled adoptively transferred TCR‐engineered T cells to effectively eradicate tumors. FPC^2^‐IG delivery system is a promising strategy for T‐cell‐based immunotherapies.

## 1 INTRODUCTION

The adoptive transfer of antigen‐specific T cells provides a promising approach for cancer treatment.^1^, ^2^ Studies have shown that the genetic modification of T cells by introduction of T‐cell receptors (TCR) or chimeric antigen receptors (CAR) has enhanced their clinical efficacy.^3^ CD19‐targeted CAR T cells have shown exceptional clinical outcomes against B cell malignancies.^4^ Clinical investigations of TCR‐T cells specific to New York esophageal squamous cell carcinoma (NY‐ESO‐1) have reported effective responses in several patients with melanoma and sarcoma.^5^, ^6^ However, the effectiveness of engineered T cells for treating solid tumors is still much lower. Unlike hematological malignancies, solid tumors generate environments that suppress antitumor immunity. In particular, low pH, limited oxygen, and the presence of suppressive cytokines (e.g., TGF‐β and IL‐10) can hamper the efficacy of adoptive T‐cell therapy (ACT).^7^ Thus, ACT for solid cancers will need to address how to improve both the persistency and potency of the engineered T cells in an immune‐suppressive tumor microenvironment.

Interleukin‐2 (IL‐2) is a cytokine that promotes T‐cell proliferation, activation, and survival.^8^, ^9^ It has been studied as an adjuvant for T‐cell‐based cancer immunotherapy. Although high‐dose bolus IL‐2 was approved by the U.S. Food and Drug Administration (FDA) for the treatment of metastatic melanoma and renal cell carcinoma, its clinical use has been hindered by its short half‐life and toxic effects, such as vascular leak syndrome, hypotension, and hepatotoxicity.^8^, ^10^, ^11^ Therefore, there is a high demand for developing IL‐2 delivery systems that can reduce the dosage of IL‐2, assure long‐lasting activity and allow localized delivery in a sustained manner.^12^

Glycosaminoglycan (GAG)‐based biomaterials are of great interest for protein delivery systems due to their unique biological merits.^13^, ^14^ Their inherent abilities to bind and sequester proteins, growth factors and cytokines via electrostatic interactions, sulfation patterns, conformations and disaccharide unit sequences often enhance protein bioactivity.^13^, ^14^ Most studies have focused on relatively small subsets of GAGs, particularly heparin due to its promiscuous‐binding ability.^13^ However, there are emerging reports that other GAGs are indeed more appropriate for the regulated delivery of specific proteins.^14^, ^15^, ^16^ Specifically, fucoidan, a GAG derived from brown algae, has shown similar or even superior binding abilities toward TGF‐β1 and IL‐6 than heparin.^15^, ^16^ Although fucoidan as a protein delivery system has not yet been trialed, it could be a promising animal‐free alternative to heparin due to its low immunogenicity, abundance in nature, high binding affinity with proteins, and anti‐tumor characteristics.^17^, ^18^

Complex coacervation is a liquid–liquid phase separation phenomenon found in natural water‐proof adhesives of marine organisms, such as mussels and sandcastle worms.^19^, ^20^ The marine organisms secrete high concentrations of water‐soluble polyelectrolytes with opposite charges. Then, the polyelectrolytes neutralize each other, or reaches electrostatic equilibrium, to form coacervates; polymer‐rich liquid droplets with size up to tens of micrometers. The coacervates act as underwater adhesives to adhere to wet surfaces or to construct protective tube‐like shelters.^19^, ^20^, ^21^ Coacervates possess extremely low interfacial energy in an aqueous solution, which enables them to engulf variety of molecules, such as artificial flavor additives and oils.^22^, ^23^ Plus, water‐immiscibility, microencapsulation ability, and biocompatibility make them an attractive source for overcoming the current drawbacks of conventional protein delivery systems.

**FIGURE 1:**
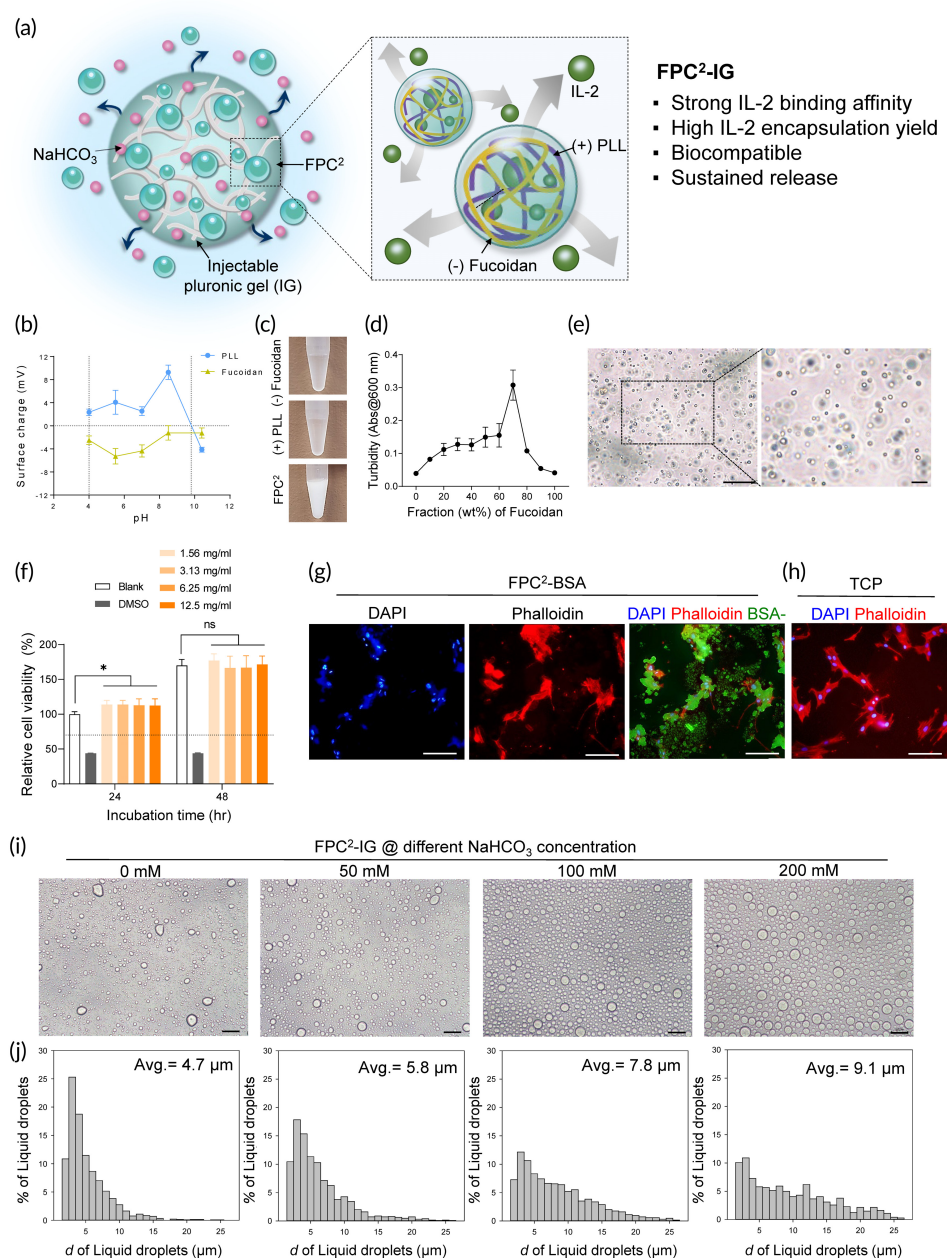
Preparation and characterization of fucoidan/PLL complex coacervate‐laden injectable hydrogel (FPC^2^‐IG). (a) Schematic overview of a FPC^2^‐IG system for effective encapsulation and sustained release of IL‐2 to enhance immunotherapy. (b) Isoelectric point detection of fucoidan and PLL by surface charge measurements (5 ≤ *n* ≤ 7). Dashed lines represent the range where net charges can be neutralized. (c) Representative photos of anionic fucoidan and cationic PLL in PBS, which instantly formed a turbid solution upon mixing. (d) Fucoidan/PLL complex coacervate (FPC^2^) formation with different weight ratios of fucoidan and PLL, as indicated by turbidity measurements (*n* = 3). (e) Optical microscopic morphology of water‐immiscible liquid droplets of FPC^2^ via liquid–liquid phase separation. Scale bar = 20 μm. (f) Human dermal fibroblasts (HDFs) viabilities after culture in medium containing FPC^2^ at different concentrations (1.56, 3.13, 6.25, and 12.5 mg/ml). Cell viabilities were normalized to that of a blank group at 24 h (*n* = 5). 15% (v/v) DMSO was used as a positive control. The red dashed line indicates a cytotoxicity threshold; a reduction in cell viability by more than 30%. Statistical significance is designated (^#^ *p* > 0.05 and *p < 0.05). (g) Fluorescent images of DAPI (blue) and phalloidin (red) staining of HDFs treated with FPC^2^ (green) after 24 h of cell culture. For visualization of FPC^2^, BSA‐FITC‐laden FPC^2^ was utilized. (h) TCP was used as a control group. Scale bar = 40 μm. (i) Optical microscopic images of FPC^2^‐IG liquid droplets with different NaHCO_3_ concentrations (0, 50, 100, and 200 mM) after incubation at 4°C for 48 h. Scale bar = 50 μm. (j) Histograms of the liquid droplet diameters under different NaHCO_3_ concentrations (0, 50, 100, and 200 mM) (*n* > 1000). All the values shown are means ± SD. Statistical significance was determined by paired *t*‐tests with two‐tailed analysis. *p < 0.05, ns: not significant

In the present study, a fucoidan‐based complex coacervate‐laden injectable hydrogel (FPC^2^‐IG) was fabricated to act as an IL‐2 delivery vehicle for enhancing ACT (Figure 1a). Fucoidan had an IL‐2‐specific binding ability, and complex coacervation was achieved through an incorporation of cationic poly‐*l*‐lysine (PLL) (FPC^2^). FPC^2^ was then embedded within pH modulating injectable gel, which was developed from the previous study^24^, to increase the stability within the acidic tumor microenvironment (TME). FPC^2^‐IG had a high IL‐2 loading efficiency, and it was able to release IL‐2 in a sustained manner in vivo. A single intratumoral administration of IL‐2‐laden FPC^2^‐IG (FPC^2^‐IG‐IL‐2) augmented the therapeutic efficacy of antitumor T cells in preclinical mouse tumor models. Flow cytometric analysis revealed that FPC^2^‐IG‐IL‐2 acted within a tumor site, which confirmed that it reduced the risk of systemic toxicity.

## 2 RESULTS

### 2.1 Preparation and characterization of fucoidan/PLL complex coacervates‐laden injectable hydrogel (FPC^2^‐IG)

Attractive electrostatic interaction is a main driving force for complex coacervation between two oppositely charged polyelectrolytes, which depends on multiple factors; such as pH, total electrolyte concentration, charge density, and salt concentration.^21^, ^22^ Before complex coacervation of fucoidan and PLL, the surface charges of each polyelectrolytes were investigated in pH ranging from 4 to 10.4 (Figure 1b). The isoelectric point of PLL deduced from surface charge measurements was pH 9.8, and fucoidan exhibited anionic surface charges at all the pH values. Therefore, complex coacervation between fucoidan and PLL was possible in the pH between 4 and 9.8; where the net charges can be mutually neutralized. In this work, a physiological condition (pH 7.4) was selected to induce complex coacervation.

Complex coacervates formed instantly, as demonstrated by turbidity increase of the mixture (Figure 1c,d), when fucoidan and PLL solutions (separately dissolved in PBS) were mixed together (FPC^2^). To determine the optimal weight ratio of fucoidan and PLL for coacervation, turbidity was measured by varying their weight ratios from 0 to 10. The maximum turbidity was recorded at a fucoidan:PLL weight ratio of 7:3, which indicated the net charge neutralization and optimal coacervation yield.^21^ In addition, the formation of spherical microdroplets, as a result of liquid–liquid phase separation, was also observed by phase‐contrast optical microscope (Figure 1e).

To assess the cytocompatibility of FPC^2^, human dermal fibroblasts (HDFs) were cultured in direct contact with different concentrations of FPC^2^ (Figure 1f). After 24 h of culture, HDFs in FPC^2^‐added groups exhibited slightly higher level of proliferation (~114 ± 6%) than the blank control group (100 ± 3%). At 72 h, cell viabilities in FPC^2^‐added groups (~177 ± 10%) were comparable to that of the control group (170 ± 9%). Furthermore, filamentous actin staining revealed that HDFs adhered on the surface with developed cytoskeletons in the presence of FPC^2^, which was comparable to the cell morphology of the control group (Figure 1g,h). These results indicated that FPC^2^ was biocompatible.

For a localized intratumoral delivery of proteins, injectable coacervate‐gel composite system with a rapid sol–gel transitional behavior was developed. FPC^2^‐IG was readily prepared by mixing of FPC^2^ suspension and pH modulating injectable hyrogel.^24^ Microscopic evaluation confirmed that NaHCO_3_ did not affect, or damage FPC^2^ liquid droplet conformation regardless of their concentration (Figure 1i). Interestingly, the average diameter of liquid droplets increased from 4.7 to 9.1 μm with increasing NaHCO_3_ concentration (Figure 1j).

### 2.2 FPC^2^ microencapsulation enables sustained and localized protein delivery with protection from proteolysis

**FIGURE 2:**
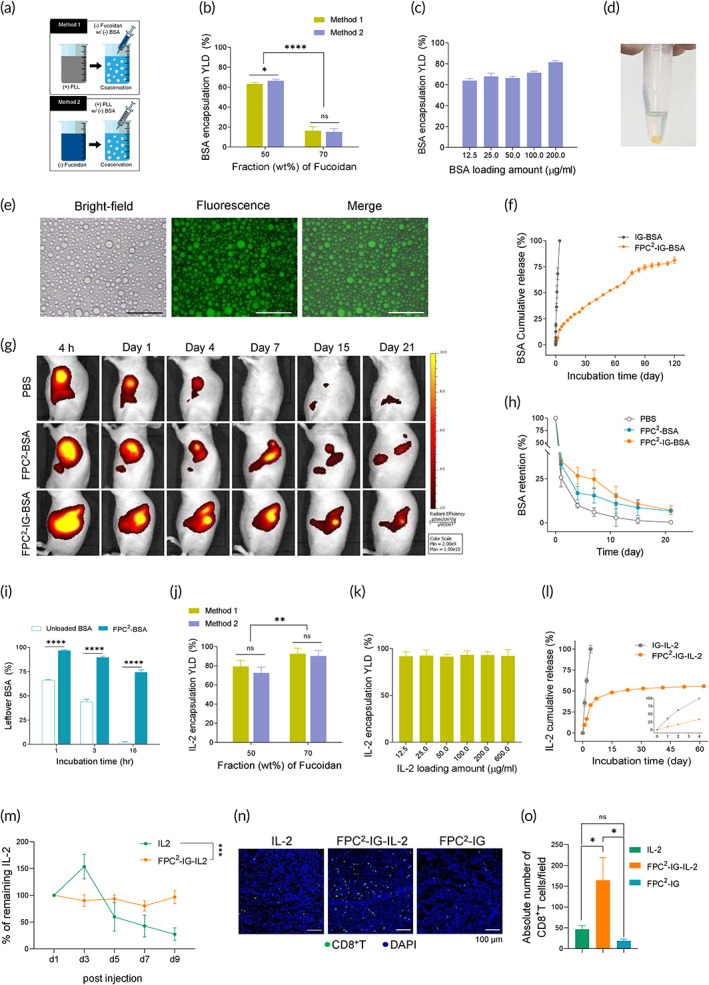
FPC^2^‐IG‐protein encapsulation abilities and release profile. (a) Schematic illustrations of BSA‐FITC encapsulation into FPC^2^ using two different mixing orders. (b) The effects of mixing order and weight ratio of fucoidan and PLL (50:50 and 70:30) on the BSA‐FITC encapsulation yields. (c) Encapsulation yields at different BSA‐FITC concentrations (12.5, 25, 50, 100, and 200 μg/ml). FPC^2^‐BSA was prepared using Method 2 with a constant fucoidan:PLL weight ratio of 50:50 (*n* = 5). (d) Representative image of a BSA‐FITC localized bulk coacervates after macro‐phase separation by centrifugation. (e) Optical microscopic, fluorescent, and merged images of FPC^2^‐IG‐BSA after thermo‐gelation at 37°C. Colocalization of green fluorescence indicates BSA‐FITC encapsulation in liquid droplets of FPC^2^. Scale bar = 50 μm. (f) In vitro release profile of BSA‐FITC from the pluronic injectable gel (IG) and FPC^2^‐IG over 120 days. Fluorescence quantification was used (*n* = 3). (g) Longitudinal IVIS images of subcutaneously injected samples containing fluorescently labeled BSA (BSA‐Vivotag 680XL) for 3 weeks using nude mice. (h) In vivo retention percentages of BSA‐Vivotag 680 XL loaded in PBS, FPC^2^, and FPC^2^‐IG by measuring the fluorescence within a constant elliptical region of interest. The fluorescent signals were normalized to day 0 values (*n* = 3). Collagen hydrogels including free and FPC^2^ encapsulated BSA‐FITC were incubated with collagenase II. (i) The leftover BSA levels were quantified by a fluorescence spectroscope at 1, 3, and 16 h after the collagenase treatment (*n* = 4). (j) Effects of the mixing order and weight ratio of fucoidan and PLL (50:50 and 70:30) on IL‐2 encapsulation yields. (k) Encapsulation yields at different IL‐2 concentrations (12.5, 25, 50, 100, 600, and 600 μg/ml). FPC^2^‐IL‐2 was prepared using Method 1 with a constant fucoidan:PLL weight ratio of 70:30 (*n* = 5). (l) In vitro release profile of IL‐2 released from IG and FPC^2^‐IG using the ELISA kit over 60 days of incubation (*n* = 3). The magnification box shows IL‐2 release profiles for the first 4 days of the incubation. (m–o) Experimental design: CT26 tumor cells were injected subcutaneously into the left flank of BALB/c mice. When the tumors reached an average volume of 100 mm^3^, free IL‐2, FPC^2^‐IG‐IL‐2, and FPC^2^‐IG were intratumorally injected into the central region of the tumors. (m) Concentrations of human IL‐2 in tumor homogenates measured by ELISA. The remaining IL‐2 level was calculated by dividing the IL‐2 concentration by its initial value on day 1. Data are representative of the two repeated experiments (*n* = 2). (n) Eight days after the injection, frozen tissue sections were stained by immunofluorescence with antibodies directed against CD8 (green) and DAPI (blue). Scale bar = 100 μm. (o) Summary graph showing the absolute number of CD8^+^ T cells per field. All the values shown are means ± SD. Statistical significance was determined by paired *t*‐tests with two‐tailed analysis. *p < 0.05, **p < 0.01, ***p < 0.001, ****p < 0.0001, ns, not significant

To evaluate protein encapsulation within FPC^2^, BSA was used as a comparative protein, since its anionic surface charges at physiological pH and high Mw (66.5 kDa) can be a contrast to IL‐2 (cationic, 15.5 kDa). Protein encapsulation was systematically studied as a function of different mixing sequences, and weight ratios of fucoidan and PLL to optimize the encapsulation conditions for BSA and IL‐2 (Figure 2a). FITC‐labeled BSA and near‐infrared fluorophore (Vivotag 680XL) labeled BSA were utilized for in vitro and in vivo studies, respectively. For BSA encapsulation, the importance of the weight ratio of fucoidan and PLL is shown in Figure 2b. Specifically, the optimal weight ratio (fucoidan:PLL = 70:30) for maximum FPC^2^ formation was not applicable for BSA encapsulation, while fucoidan:PLL ratio of 50:50 (wt%) showed higher encapsulation yields than 70:30 (wt%) ratio; ~66.4 ± 1.7%, ~16.3 ± 4.0%, respectively. The effect of mixing sequences on encapsulation yields was not significant. However, the formation of an intermediate complex consisting of BSA and PLL (Method 2), where different surface charges are mixed prior to coacervation, led to slightly higher encapsulation yields (63.2 ± 1.7% and 66.4 ± 1.7% for Method 1 and Method 2), at a 50:50 (wt%) ratio. Furthermore, the encapsulation efficiency with different BSA concentrations, ranging from 12.5 μg/ml to 200 μg/ml, using Method 2 and the optimal fucoidan:PLL weight ratio (50:50) is shown in Figure 2c. As BSA concentration increased, encapsulation yields increased up to ~81.5 ± 1.5%. The successful encapsulation of BSA was also visually confirmed by a yellow‐colored sediment of bulk coacervates after centrifugation (Figure 2d). Additionally, colocalization of green fluorescence from the BSA within FPC^2^ liquid droplets was observed after incubation with a pluronic prehydrogel solution (Figure 2e).

BSA release profile from FPC^2^ was assessed by incubation in PBS. Nearly 100% release was observed after 4 days of incubation for the IG group due to the fast dissolution of pluronic gel (Figure 2f).^25^ In contrast, 14.4% of BSA was released from FPC^2^‐IG in a sustained manner over the same period of incubation time. Particularly, FPC^2^‐IG was able to release BSA without sudden outbursts with an overall cumulative release of 81.4% for 120 days of incubation. Furthermore, to evaluate BSA retention in vivo, subcutaneously injected samples with Vivotag 680XL labeled BSA was tracked using IVIS spectra (Figure 2g). As indicated by the fluorescence areas after 4 h of injection, FPC^2^ and FPC^2^‐IG potentiated the initial retention of protein at the injection site compared to that of the PBS injection, since the water‐immiscibility of FPC^2^ (Figure S1) and rapid thermo‐gelation of IG stopped BSA from washing off. In both of FPC^2^ and FPC^2^‐IG groups, BSA was observed after 3 weeks of injection, while fluorescence was rarely detected after 1 week for the PBS group. BSA retention rates (%) were also quantified by normalizing their fluorescent signals to 4 h time point values (Figure 2h). After 1 week, BSA retention yield for the FPC^2^‐IG group was 20% higher than that of the PBS group and 10% higher than that of the FPC^2^ only group, indicating prolonged BSA retention. After 3 weeks, FPC^2^ and FPC^2^‐IG exhibited similar BSA retentions (6.7% and 7.2%), and there was no fluorescence detection for the PBS group.

To evaluate the protein protection abilities of FPC^2^ from enzymatic degradation, free BSA, and BSA‐laden FPC^2^ were loaded into collagen prehydrogel solution, which served as an extracellular matrix. After the gelation of collagen, collagenase was added to the gels to mimic the tumor microenvironment; where matrix metalloproteinases are prominent.^26^ Collagenase was able to disassemble not only the collagen gel but also FPC^2^ via PLL degradation. As shown in Figure 2i, after 3 h of postincubation, only 43.9 ± 2.6% of the unprotected BSA remained, while microencapsulated BSA within FPC^2^ was significantly protected from the proteolysis; nearly 2‐fold (89.7 ± 1.3%) higher than the unprotected group. After 16 h, unprotected BSA was hardly detected, whereas 74.4 ± 2.5% of the initial BSA remained within FPC^2^. These results were also verified by a visual observation after centrifugation of the lysates (Figure S2). Fluorescent and optical images showed colocalization of BSA in microcapsules of FPC^2^, implying that FPC^2^ can encapsulate and protect proteins from proteolysis and prolong their stability in vivo.

Following the same procedure as BSA encapsulation, IL‐2 encapsulating conditions were optimized by evaluating different mixing sequences and weight ratios of fucoidan and PLL (Figure S3). On the contrary to BSA encapsulation, IL‐2 showed higher encapsulation yields at the fucoidan:PLL ratio of 7:3 (wt%) (~92.5 ± 6.0%), the expected optimal coacervation yield, compared to 5:5 (wt%) ratio (~79.3 ± 6.4%). As for the mixing sequences, there were no significant statistical differences. The IL‐2 concentrations on encapsulation yields did not have significant effects as well (Figure 2k). IL‐2 concentrations ranging from 12.5 to 600 μg/ml confirmed more than 91.5% encapsulation.

In vitro release profile of IL‐2 from IG (IG‐IL‐2) and FPC^2^‐IG (FPC^2^‐IG‐IL‐2) was assessed by ELISA (Figure 2l). Similar to the BSA release profile, IG‐IL‐2 released 100% of the encapsulated IL‐2 within 4 days of incubation. In contrast, only one‐third of the initial IL‐2 was released after 4 days (33.2 ± 1.8%) of incubation for FPC^2^‐IG‐IL‐2. Additionally, the cumulative release measurement for 62 days was 55.8 ± 0.03% of the total encapsulated IL‐2.

The effect of FPC^2^ on IL‐2 protection and sustainability in vivo was assessed by measuring the IL‐2 concentration within tumors. Mice bearing CT26 tumors were intratumorally injected with free IL‐2 and FPC^2^‐IG‐IL‐2. Then, the levels of IL‐2 from tumor homogenates were measured over 9 days. While the IL‐2 level dropped by approximately 50% after 5 days of injection for the free IL‐2 injected group, the IL‐2 level was maintained over 9 days for the FPC^2^‐IG‐IL‐2 injected group (Figure 2m). To confirm the sustainability of IL‐2 by FPC^2^ encapsulation in vivo, CD8^+^ T‐cell infiltration into tumors after a single intratumoral injection of FPC^2^‐IG‐IL‐2 was examined. As shown in Figure 2n and o, the immunofluorescence staining of tumor tissue sections revealed that tumor‐infiltrating CD8^+^ T cells in CT26‐bearing mice with FPC^2^‐IG‐IL‐2 injection was 3‐fold higher than those injected with FPC^2^‐IG only or with free IL‐2. The superior activity of FPC^2^‐IG‐IL‐2 was further confirmed by comparing to the groups that received multiple doses of free IL‐2 via *i*.*t*. or *i*.*p*. route (Figure S4). This indicated that the FPC^2^ encapsulation facilitates IL‐2 protection in TME, which could promote the infiltration and proliferation of CD8^+^ T cells in the TME.

### 2.3 Microencapsulation improves IL‐2 bioactivity toward effector T cells

**FIGURE 3:**
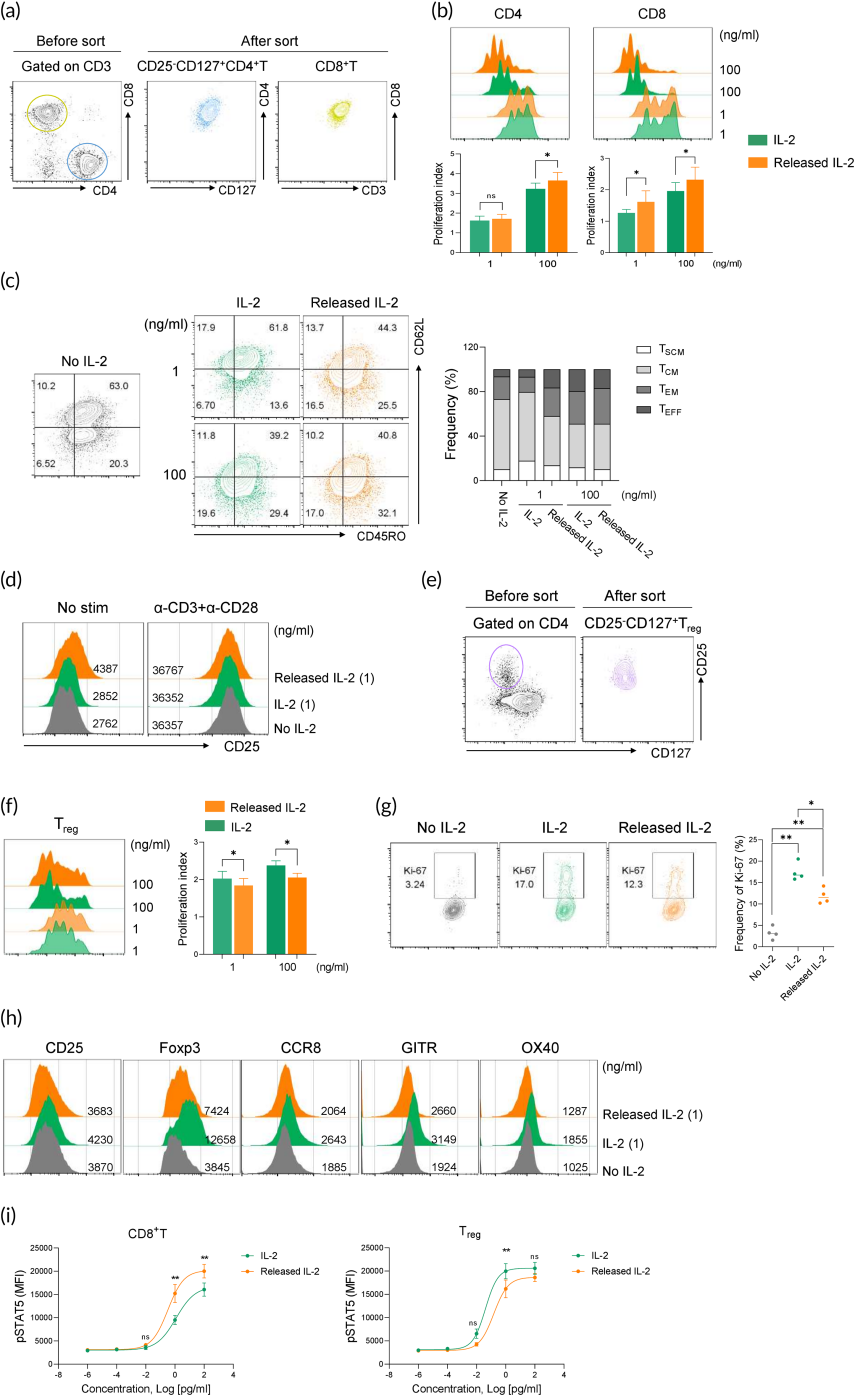
The microencapsulation of IL‐2 by FPC^2^ shows bioactivity toward effector T cells over Treg cells. (a) FACS sorting strategy (left) and post‐sorting purity (right) in relation to CD25^−^CD127^+^CD4^+^ and CD8^+^T cells isolated from human PBMCs. (b) Histogram plots showing the CTV dilution (above) and bar graphs showing the proliferation index (below) of CD4^+^T and CD8^+^T cells upon stimulation with anti‐CD3/CD28 antibodies in the presence of free IL‐2 and FPC^2^‐IG‐IL‐2 at final concentrations of either 1 or 100 ng/ml for 5 days. (c) Representative FACS plots (left) and summary bar graphs (right) showing human CD8^+^T cell memory differentiation in the presence of free IL‐2 and FPC^2^‐IG‐IL‐2 at final concentrations of either 1 or 100 ng/ml for 5 days. (d) Histogram plots showing CD25 expression by CD8^+^T cells with and without anti‐CD3/CD28 stimulation in the presence of free IL‐2 and FPC^2^‐IG‐IL‐2 at a final concentration of 1 ng/ml for 3 days. (e) FACS sorting strategy (left) and post‐sorting purity (right) for CD25^+^CD127^−^CD4^+^ Treg cells that were isolated from human PBMCs. (f) Histogram plots showing CTV dilution (left) and bar graphs showing the proliferation index (right) of Treg cells upon stimulation with anti‐CD3/CD28 antibodies in the presence of free IL‐2 and FPC^2^‐IG‐IL‐2 at a final concentration of 1 and 100 ng/ml for 5 days. (g‐h) FACS‐sorted Treg cells were incubated in the presence of free IL‐2 and FPC^2^‐IG‐IL‐2 at a final concentration of 1 ng/ml for 3 days. (g) Representative FACS plots (left) and a summary plot (right) showing Ki‐67 expression by Treg cells. (h) Histogram plots showing the geometric mean fluorescence intensity of the indicated marker expression levels in Treg cells. (i) Phospho‐flow staining of STAT5 in CD8^+^T (left) or Treg cells (right) in the presence of free IL‐2 and FPC^2^‐IG‐IL‐2 at various concentrations. Data were pooled from three independent experiments. Statistical significance was determined by paired *t*‐tests with two‐tailed analysis in (b), (f) and (g) or one‐way ANOVA with Holm–Sidak multiple comparisons in (i). *p < 0.05, **p < 0.01, and ns: not significant

After confirming the encapsulation ability of FPC^2^, immune cell viabilities, or nonspecific immune activation properties, of fucoidan and FPC^2^ were evaluated with human PBMCs. Neither fucoidan nor FPC^2^ induced aberrant immune cell death and activation, or further promoted the proliferation of particular immune cell types (Figure S5). To examine the bioactivity of IL‐2 released from FPC^2^, CD25^−^CD127^+^CD4^+^ (Treg negative) and CD8^+^T cells were FACS‐sorted from the human PBMCs (Figure 3a), and then stimulated with anti‐CD3 and anti‐CD28 antibodies in the presence of free IL‐2 or FPC^2^‐IG‐IL‐2. FACS‐sorted CD4^+^ and CD8^+^ effector T cells showed higher proliferative capacities upon treatment with FPC^2^‐IG‐IL‐2 compared to that of the free IL‐2 treatment at both low and high concentrations (Figure 3b).

A previous study has shown that introducing a low‐concentration of IL‐2 increased the abundance of CD62L^+^ stem cell memory (T_SCM_) and central memory (T_CM_) T‐cell populations, whereas strong IL‐2 signaling induced effector memory (T_EM_) or effector (T_EFF_) CD8^+^T cell differentiation.^27^ CD8^+^T cell memory subsets were assessed to confirm whether FPC^2^‐IG‐IL‐2 conferred a higher bioactivity than the free IL‐2 on CD8^+^T cells. A low concentration of released IL‐2 from FPC^2^‐IG‐IL‐2 promoted CD62L^−^ T_EM_ and T_EFF_ cell generation while T_SCM_ population was enriched by the free IL‐2 treatment. At a high concentration, both free IL‐2 and FPC^2^‐IG‐IL‐2 induced the downregulation of CD62L and the enrichment of T_EM_ and T_EFF_ subsets (Figure 3c).^27^ The higher responsiveness of CD8^+^T cells to FPC^2^‐IG‐IL‐2 was further exhibited by an increased expression of CD25 (IL‐2 receptor α) as compared to free IL‐2, which did not upregulate CD25 under a steady‐state condition (Figure 3d). However, upon TCR/CD28 stimulation, a comparable level of CD25 expression was found in between FPC^2^‐IG‐IL‐2 and free IL‐2 treated CD8^+^T cells, which suggested that FPC^2^‐IG‐IL‐2 induced a strong signal through the IL‐2 signaling pathway to modulate CD8^+^T cell responses without affecting TCR‐induced T‐cell activation.

IL‐2‐induced Treg cell expansion has been a major drawback for IL‐2‐based cancer immunotherapies. Therefore, the effect of FPC^2^‐IG‐IL‐2 on Treg cell expansion was compared to that of the free IL‐2 treatment on the proliferative response. FACS‐sorted CD25^+^CD127^−^CD4^+^Treg cells exposed to FPC^2^‐IG‐IL‐2 showed reduced proliferative capacities than the cells treated with free IL‐2 at both the low and high concentrations (Figure 3e,f). Both frequencies and expression levels of Ki‐67 were also decreased in Treg cells with FPC^2^‐IG‐IL‐2 treatment (Figure 3g). In addition, FPC^2^‐IG‐IL‐2 was less effective at upregulating Treg cell markers, including CD25, Foxp3, CCR8, GITR, and OX40, which represented effector Treg cells with strong suppressor functions (Figure 3h).^28^ The biased bioactivity of FPC^2^‐IG‐IL‐2 toward CD8^+^T cells was further confirmed by phospho‐flow staining of STAT5 (pSTAT5), which is a key downstream indicator of IL‐2R signaling. FPC^2^‐IG‐IL‐2 more efficiently promoted STAT5 phosphorylation in CD8^+^T cells compared to free IL‐2, but not in Treg cells (Figure 3i). Collectively, these results suggested that the IL‐2 released by FPC^2^‐IG could preferentially promote effector T‐cell proliferation over Treg cells.

### 2.4 Modulation of intratumoral immune cell responses by FPC^2^‐IG‐IL‐2

The impact of IL‐2 expression on the patient survival with lung adenocarcinoma and skin cutaneous melanoma in TCGA using the Kaplan–Meier survival analysis was investigated. Patients with high levels of IL‐2 expression experienced longer overall survival (OS) (Figure S6a). To evaluate the good prognosis of lung adenocarcinoma (LUAD) and skin cutaneous melanoma (SKCM) with high IL‐2 expression, infiltration of antitumor immune cells within the tumor sites were examined. As shown in Figure S6b, there were positive correlations between IL‐2 expression, CD8^+^ T, and Treg cells infiltrations. The positive prognostic effect of Treg cells was possibly due to the concomitant abundance of CD8^+^ T‐cell infiltration.

**FIGURE 4:**
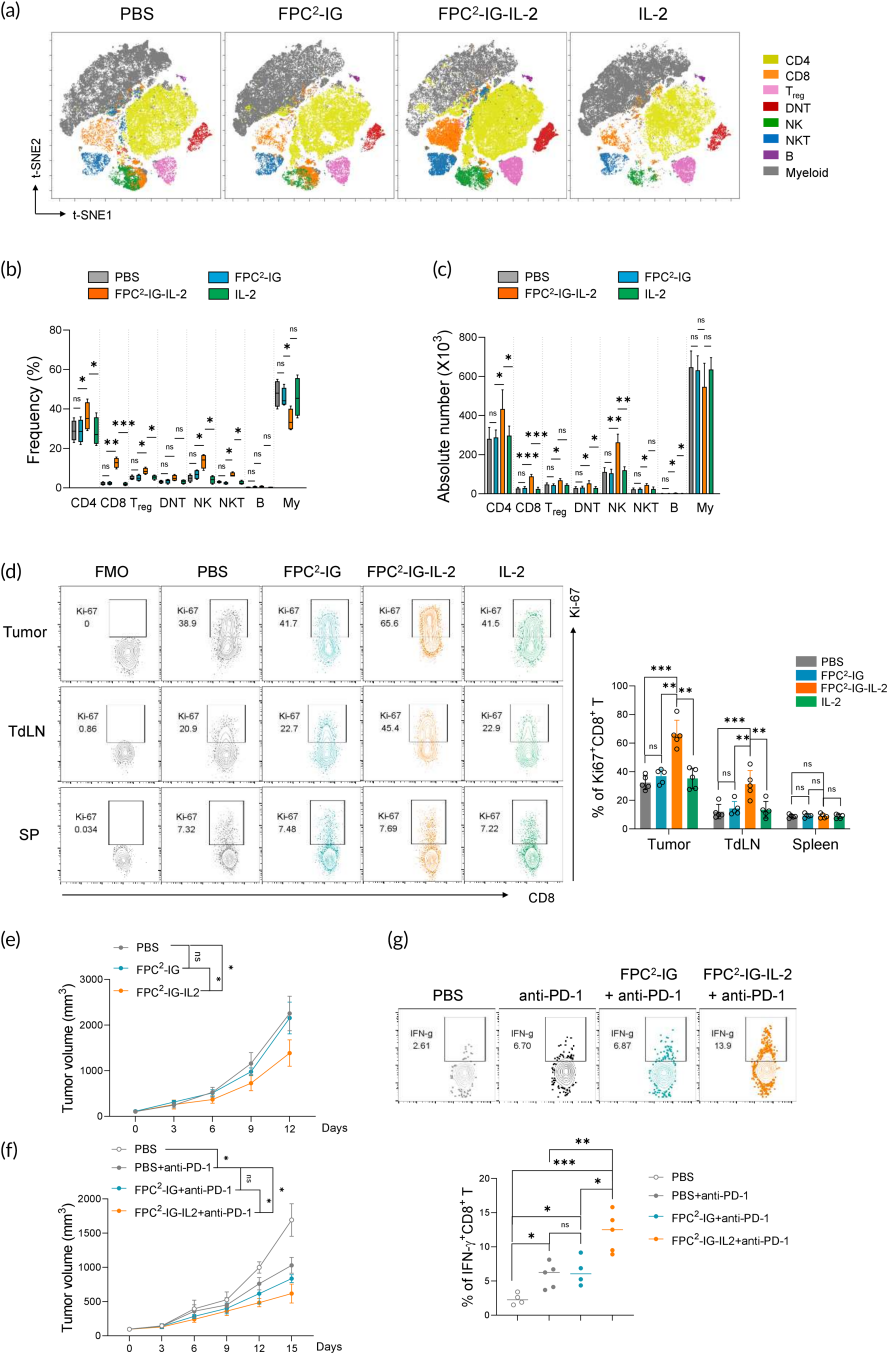
Enhancement of antitumor immune responses by FPC^2^‐IG‐IL‐2. (a–e) Experimental design: CT26 tumor cells were injected subcutaneously into the left flank of BALB/c mice. When the tumors reached an average volume of 50 mm^3^, FPC^2^‐IG‐IL‐2, FPC^2^‐IG, or free IL‐2 were intratumorally injected into the tumors. (a) Representative *t*‐SNE plots showing immune cells within CT26 tumors. (b) Summary graphs showing the frequencies of the annotated clusters. (c) Summary graphs showing the absolute numbers of the annotated clusters. (d) Representative flow cytometric plots showing the expression of Ki‐67 expression in CD8^+^ T cells within tumors, a tumor‐draining lymph node (TdLN), and the spleen (SP) (left). A summary graph showing the percentages of Ki‐67 expression in tumor‐infiltrating CD8^+^ T cells (right). (e) Changes in mean tumor volume over time. (f,g) Experimental design: CT26 tumor cells were implanted into the left flank of BALB/c mice, and treatment was started when the tumor reached a size of approximately 75 mm^3^. The mice were then treated with PBS, FPC^2^‐IG, and FPC^2^‐IG‐IL‐2 followed by anti‐PD‐1 antibody (200 μg; on day 5, 8, and 11) administration after 3 days of the injections. Data represent tumor volume ± SEM; *n* = 5 mice per group. (f) Changes in mean tumor volume over time. (g) Flow cytometric analysis of the expression of IFN‐γ by tumor‐infiltrating AH1‐specific CD8^+^ T cells stimulated with AH1 peptide in the presence of Brefeldin A (above). A summary graph showing the frequencies of IFN‐γ expressions by CD8^+^ T cells in CT26 tumors (*n* = 4–5) (below). Statistical significance was determined by paired *t*‐tests with two‐tailed analysis in (b–d) and (g) or two‐way ANOVA with Tukey's multiple comparisons in (e) and (f). *p < 0.05, **p < 0.01, ***p < 0.001, ns: not significant

The effects of FPC^2^‐IG‐IL‐2 on tumor‐infiltrating immune cell populations were examined by the immune monitoring of CT26‐bearing mice with intratumorally injected PBS, FPC^2^‐IG, FPC^2^‐IG‐IL‐2, and free IL‐2. Ten days after the injection, the CT26 tumor was prepared as a single cell suspension and analyzed by multi‐color flow cytometry. The distribution of eight immune cell subsets was visualized in a *t*‐stochastic neighbor embedding (*t*‐SNE) map (Figure 4a and Figure S7). These subsets were CD4^+^T, CD8^+^T, Treg, CD4, and CD8 double‐negative (DN) T, NK, NKT, B, and myeloid cells; manually annotated. FPC^2^‐IG‐IL‐2 treatment led to an increase in lymphocyte populations compared to other injections, while myeloid populations were decreased (Figure 4b). Among the lymphocyte subpopulations, both the frequencies and numbers of cytotoxic lymphocytes, including CD8^+^T and NK cells, were significantly increased compared to CD4^+^T, Treg, DNT, NKT, and B cells (Figure 4b,c). These results suggested that FPC^2^‐IG‐IL‐2 alters the tumor‐immune microenvironment (TIME) by preferentially facilitating cytotoxic lymphocyte proliferation. Indeed, CD8^+^T cells derived from tumors and tumor‐draining lymph nodes (TdLN) had a higher frequency of Ki‐67 expression in CT26‐bearing mice with FPC^2^‐IG‐IL‐2 injection compared to those that received PBS, FPC^2^‐IG, and free IL‐2 (Figure 4d). However, splenic CD8^+^T cells showed similar levels of Ki‐67 expression for all the sample groups, which confirmed that FPC^2^‐IG‐IL‐2 was precisely localized within tumors. Despite FPC^2^‐IG‐IL‐2 induced enrichment of tumor‐infiltrating cytotoxic lymphocytes, FPC^2^‐IG‐IL‐2 injection alone did not induce a sufficient antitumor immune response against CT26 tumors. As shown in Figure 4e, there was only a moderate degree of tumor growth inhibition observed in CT26‐bearing mice with FPC^2^‐IG‐IL‐2 injection. Then, to examine whether FPC^2^‐IG‐IL‐2 could potentiate the antitumor efficacy of PD‐1 blockade, anti‐PD‐1 antibody was administered to CT26‐bearing mice at 3 days after single PBS, FPC^2^‐IG, or FPC^2^‐IG‐IL‐2 intratumoral injection. A significant regression of tumor growth was exerted by an anti‐PD‐1 antibody treatment in the CT26‐bearing mice receiving FPC^2^‐IG‐IL‐2 compared to those receiving PBS or FPC^2^‐IG (Figure 4f). The enhanced antitumor immune response caused by the combination of FPC^2^‐IG‐IL‐2 and anti‐PD‐1 antibody was further demonstrated in tumor‐infiltrating CD8^+^T cells that exhibited a higher frequency of IFN‐γ expression upon stimulation with AH1 peptide, the immunodominant antigen of CT26 (Figure 4g). These results suggested that antitumor immunity can be enhanced by a single intratumoral injection of FPC^2^‐IG‐IL‐2 in a localized manner.

### 2.5 Enhanced intratumoral expansion and antitumor responses of adoptively transferred tumor‐reactive T cells by FPC^2^‐IG‐IL‐2

**FIGURE 5:**
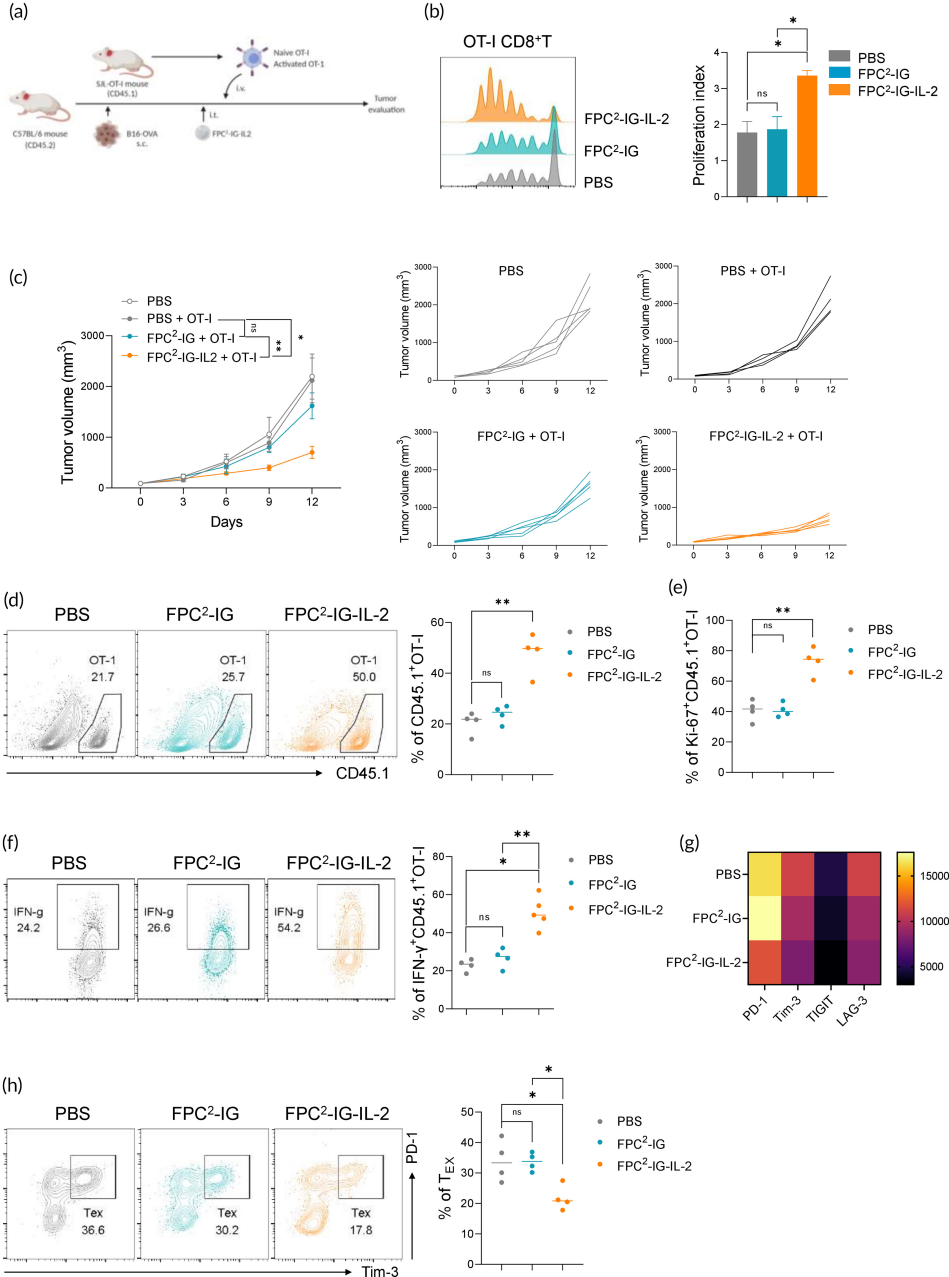
FPC^2^‐IG‐IL‐2 promotes the antitumor responses of adoptively‐transferred CD8^+^ T cells. (a–h) Experimental design: 2 × 10^5^ B16‐OVA tumor cells were injected subcutaneously into the left flank of C57BL/6 mice on day 0 (*n* = 5). PBS, FPC^2^‐IG, or FPC^2^‐IG‐IL‐2 were then intratumorally injected into the central region of the tumors on day 7. One day after these injections, naïve (b), and activated (c–h) CD45.1^+^ OT‐I T cells were adoptively transferred intravenously into the tumor‐bearing mice. (b) Representative flow cytometric plots (left) showing the CTV dilution of tumor‐infiltrating CD45.1^+^ OT‐I T cells, and a bar graph (right) summarizing the proliferation index. Experiments were conducted twice with two mice per group. (c) Splenocytes isolated from CD45.1^+^ OT‐I mice were activated with SIINFEKL peptide. CD8^+^ T cells were purified and adoptively transferred into the mice bearing B16‐OVA tumors. Average tumor sizes for all of the tumor growth curves per group (left) and individual mice (right) are indicated. (d) Representative flow cytometric plots (left) and a summary plot (right) showing the percentages of CD45.1^+^ OT‐I T cells in tumors. (e) Summary graph showing the percentages of Ki‐67 expression in tumor‐infiltrating CD45.1^+^ OT‐I T cells. (f) Representative flow cytometric plots (left) and a summary plot (right) showing the percentages of IFN‐γ expression by tumor‐infiltrating CD45.1^+^ OT‐I T cells. (g) Heat map plot showing the geometric mean fluorescence intensity of the expression indicated by immune checkpoint receptors in tumor‐infiltrating CD45.1^+^ OT‐I T cells. (h) Representative flow cytometric plots (left) and a summary plot (right) showing the percentages of exhausted CD45.1^+^ OT‐I T cells (PD‐1+Tim‐3+) within tumors. Statistical significance was determined by paired *t*‐tests with two‐tailed analysis in (b), (d), (e), (f), (h), or two‐way ANOVA with Tukey's multiple comparisons in (c). *p < 0.05, **p < 0.01, ns, not significant

After confirming the ability of FPC^2^‐IG‐IL‐2 to enhance the therapeutic efficacy of anti‐PD‐1 in preclinical mouse models, the antitumor effects of FPC^2^‐IG‐IL‐2 in support of the expansion, and functionality of adoptively transferred T cells were examined. C57BL/6 mice bearing B16‐OVA melanomas were intratumorally administered with FPC^2^‐IG and FPC^2^‐IG‐IL‐2, followed by the adoptive transfer of congenially distinguishable naïve tumor‐reactive T cells (CD45.1^+^OT‐I; Figure 5a). To monitor the trafficking of the transferred T cells, CD8^+^ T cells isolated from CD45.1^+^ OT‐I mice, on which the TCR can recognize OVA peptides in the context of H‐2K^b^, were labeled with CTV and intravenously transferred into the C57BL/6 mice (CD45.2) bearing B16‐OVA tumors. The proliferation of the transferred naïve OT‐I T cells was significantly increased in the FPC^2^‐IG‐IL‐2 treated group, as revealed by measuring the dilution of CTV fluorescent intensity (Figure 5b). This indicated that the released IL‐2 from FPC^2^‐IG‐IL‐2 can efficiently support the expansion of tumor‐infiltrating, adoptively transferred, tumor‐reactive T cells. The antitumor efficacy of the OT‐I + FPC^2^‐IG‐IL‐2 combination was assessed. As shown in Figure 5c, OT‐I + FPC^2^‐IG‐IL‐2 combination treatment significantly enhanced tumor growth inhibition compared to mice that received OT‐I alone or the OT‐I + FPC^2^‐IG combination. In the tumors of the FPC^2^‐IG‐IL‐2 treated mice, the infiltration and expansion of adoptively transferred OT‐I T cells were increased (Figure 5d,e). Ki‐67 expression was used for the measurement of proliferating OT‐I T cells (Figure 5e). The functional properties of the transferred T cells between the FPC^2^‐IG and FPC^2^‐IG‐IL‐2 treated mice were compared. CD45.1^+^OT‐I T cells from the mice treated with OT‐I + FPC^2^‐IG‐IL‐2 produced higher levels of IFN‐γ compared to those in the mice that were given OT‐I alone or an OT‐I + FPC^2^‐IG combination (Figure 5f). Tumor‐infiltrating CD8^+^ T cells progressively became dysfunctional, or exhausted, upon binding with their cognate antigens. Exhausted CD8^+^ T cells exhibited high co‐expression of inhibitory receptors; including PD‐1, TIGIT, lymphocyte‐activation gene 3 (LAG‐3), and T‐cell immunoglobulin and mucin‐domain containing‐3 (Tim‐3). Flow cytometric analysis of tumor‐infiltrating T cells revealed a decrease in the frequency of PD‐1^+^Tim‐3^+^ CD45.1^+^ OT‐I T cells in the FPC^2^‐IG‐IL‐2 treated group. This indicated that FPC^2^‐IG‐IL‐2 administration can reduce the exhaustion of adoptively transferred tumor‐reactive T cells (Figure 5g,h). Taken together, these results confirmed that local tumor treatment with FPC^2^‐IG‐IL‐2 can enhance the proliferation and activation of adoptively transferred tumor‐reactive CD8^+^ T cells, which leads to improved antitumor control.

### 2.6 FPC^2^‐IG‐IL‐2 potentiates the antitumor efficacy of TCR‐engineered T‐cell therapy

**FIGURE 6:**
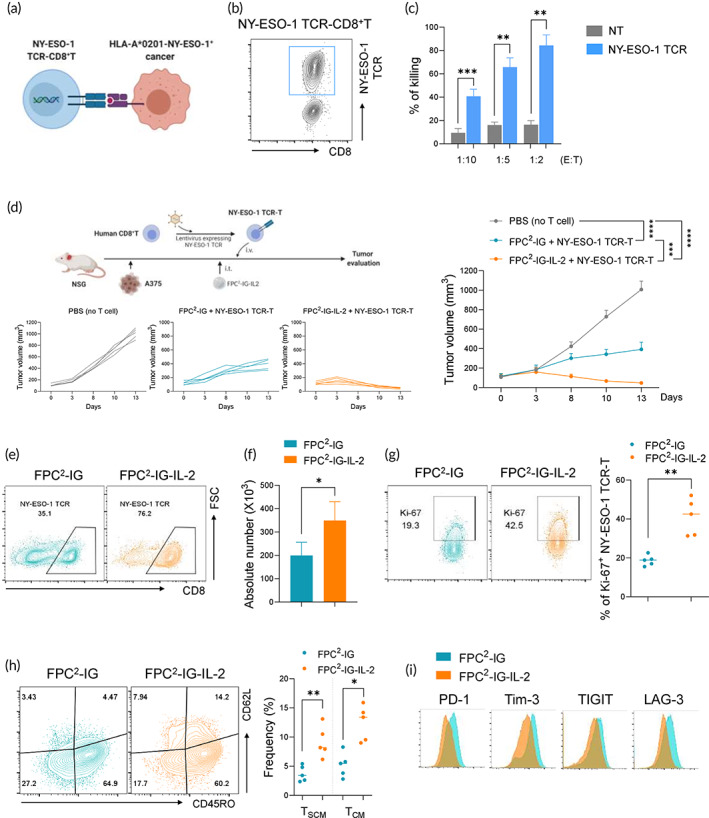
Enhancement of antitumor efficacy of NY‐ESO‐1 TCR‐T by intratumoral administration of FPC^2^‐IG‐IL‐2. (a) Schematic illustration of NY‐ESO‐1 TCR‐T cell‐mediated target cell cytotoxicity. (b) Flow cytometric analysis of the expression of NY‐ESO‐1 TCR on human CD8^+^ T cells. (c) NY‐ESO‐1 TCR and nontransduced (NT) T cells were incubated with A375‐LUC cells for 24 h. The tumor cell cytotoxicity was then determined by the loss of luciferase activity. (d–i) Experimental design: 1 × 10^6^ A375 tumor cells were injected subcutaneously into the left flank of NSG mice at day 0 (*n* = 5). PBS, FPC^2^‐IG, or FPC^2^‐IG‐IL‐2 were intratumorally injected into the tumors at day 7. One day after these injections, NY‐ESO‐1 TCR‐T cells were intravenously adoptively transferred into the tumor‐bearing mice. (d) Average tumor sizes determined from all of the tumor growth curves per group (right) and in individual mice (left). (e) Representative flow cytometric plots showing the frequencies (e) and a bar graph showing absolute numbers (f) of NY‐ESO‐1 TCR‐T cells in the tumors. (g) Representative flow cytometric plots (left) and a summary plot (right) of Ki‐67 expression in tumor‐infiltrating NY‐ESO‐1 TCR‐T cells. (h) Representative flow cytometric plots (left) and a summary plot (right) showing T‐cell subsets in tumor‐infiltrating NY‐ESO TCR‐T cells, that is, T_CM_ (CD62L^+^CD45RO^+^), T_EM_ (CD62L^−^CD45RO^+^) and T_EMRA_ (CD62L^−^CD45RO^−^). (i) Flow cytometric analysis showing the expression of inhibitory checkpoint receptors on tumor‐infiltrating NY‐ESO TCR‐T cells. Statistical significance was determined by paired *t*‐tests with two‐tailed analysis in (c), (f), (g), (h), or two‐way ANOVA with Tukey's multiple comparisons in (d). *p < 0.05, **p < 0.01, ***p < 0.001, ****p < 0.0001, ns, not significant

To assess whether our current study findings could be translated into possible therapeutic applications, the antitumor activity of an engineered human T‐cell + FPC^2^‐IG‐IL‐2 combination was explored. Primary human T cells were genetically modified with a recombinant TCR specific for the tumor antigen NY‐ESO‐1. NY‐ESO‐1 TCR‐expressing CD8^+^T cells can specifically recognize a NY‐ESO‐1 epitope (157–165) expressed on cancer cells in an HLA‐A*0201‐restricted manner (Figure 6a,b). In vitro co‐culture of A375 cancer cells (HLA‐A*0201+, NY‐ESO‐1+) with NY‐ESO‐1 TCR‐CD8^+^T cells led to the efficient killing of A375 cells in a dose‐dependent manner (Figure 6c). NSG mice were implanted with A375 cells and intratumorally treated with PBS, FPC^2^‐IG, and FPC^2^‐IG‐IL‐2. Then, NY‐ESO‐1 TCR‐CD8^+^T cells were adoptively transferred intravenously into the NSG mice at 5 days after the intratumoral injection. A superior antitumor response by adoptively transferred NY‐ESO‐1 TCR‐CD8^+^T with FPC^2^‐IG‐IL‐2 was observed. The tumor volume was significantly reduced by 95.3% and 88% in comparison to the PBS and FPC^2^‐IG injections, respectively (Figure 6d). Flow cytometric analysis revealed that FPC^2^‐IG‐IL‐2 administration induced an increase in the expansion of NY‐ESO‐1 TCR‐T cells within tumors (Figure 6e,f). Ki‐67 expression marker analysis showed that FPC^2^‐IG‐IL‐2 treatment led to an increase in proliferation of NY‐ESO‐1 TCR‐T cells (Figure 6g). Previous studies have shown that the replication and survival capacity of adoptively transferred T cells correlates with their antitumor efficacy. T_SCM_, one of the subsets of human T cells, has enhanced proliferation and survival capabilities compared to other subsets. Analysis of tumor‐infiltrating NY‐ESO‐1 TCR‐T cells revealed that the frequencies of T_SCM_ and T_CM_ were higher in the FPC^2^‐IG‐IL‐2 than FPC^2^‐IG treated groups (Figure 6h). Tumor‐infiltrating NY‐ESO‐1 TCR‐T cells were profiled via inhibitory immune checkpoint receptors that mark the exhausted state of CD8^+^ T cells. NY‐ESO‐1 TCR‐T cells that infiltrated the tumors of FPC^2^‐IG‐IL‐2 treated mice exhibited a decreased expression of PD‐1, Tim‐3, TIGIT, and LAG‐3, which is an indicative phenotypes of less exhausted T cells (Figure 6i). These data suggested that FPC^2^‐IG‐IL‐2 treatment can potentially increase the antitumor effects of NY‐ESO‐1 TCR‐T cells by maintaining self‐renewing, less exhausted, and antigen‐reactive T cells.

## 3 DISCUSSION

With the recent encouraging results from engineered IL‐2 anticancer therapies in preclinical animal models and humans, various approaches to improve the therapeutic efficacy and safety of IL‐2 have been attempted. In this study, a coacervate‐hydrogel hybrid system was designed; IL‐2‐ladened FPC^2^ embedded in the injectable hydrogel (FPC^2^‐IG‐IL‐2), to localize the effects of IL‐2 and prolong IL‐2 delivery in tumors.

To date, coacervation has been mainly studied as adhesives in bulk forms,^29^ and encapsulating molecules and cells (i.e., stem cells).^23^, ^30^ Although bulk coacervates and coacervate suspensions (liquid droplets) are reported to have similar diffusion coefficients,^22^ molecule release rate of the bulk coacervates is inferior due to their low surface area to volume ratio. From that respect, liquid micro‐droplet conformation of coacervates was hypothesized to be more ideal for an effective protein delivery system.

Polyelectrolyte selection was an important factor for designing a stable complex coacervate, because intensive electrostatic interactions between strongly charged polyelectrolytes tend to cause precipitation (liquid–solid separation) rather than coacervation (liquid–liquid separation).^21^ In this study, PLL was selected as a positive polyelectrolyte, since it is an FDA‐approved natural homopolypeptide which can be manufactured with controlled molecular weights and low polydispersity.^31^ An optimal anionic counterpart was required to have a strong IL‐2 binding ability for sustained IL‐2 release. Therefore, three different sulfated polysaccharides; heparin, chondroitin sulfate A (CS‐A), and fucoidan, were assessed via sandwich ELISA (Figure S8). IL‐2 had a preferential binding affinity toward both heparin and fucoidan, whereas CS‐A, a monosulfated GAG,^32^ exhibited negligible binding affinity with IL‐2. Although heparin showed slightly higher binding affinity with IL‐2 than fucoidan, more than 1 M concentrated salt (NaCl) was required for complex coacervation between PLL and heparin to alleviate their strong electrostatic interaction (Figure S9), which was not ideal for the intratumoral injection. Interestingly, the combination of fucoidan and PLL (FPC^2^) led to an increase in relative binding abilities to IL‐2, which was also comparable to that of heparin.

The water‐immiscibility and strong IL‐2 binding affinity of FPC^2^ allowed sustained delivery of IL‐2 into tumors with just a single injection. In addition, we were able to demonstrate that FPC^2^‐mediated microencapsulation provided encapsulated proteins with an effective protection against enzymatic degradation and prolongation of their bioactivity. These characteristics of FPC^2^‐IG‐IL‐2 could significantly diminish the issues presented by the current IL‐2 therapy with high‐dose IL‐2, which includes toxicity and a short half‐life. Prior reports have shown that a systemic administration of high‐dose IL‐2 is associated with treatment‐related mortality, including capillary leak syndrome in multiple organs.^33^ This can be mitigated by a localized intratumoral injection with sustained IL‐2 delivery.^11^ The present study confirmed that treatment via intratumoral injection of FPC^2^‐IG‐IL‐2 increases Ki‐67 expression by CD8^+^T cells within tumors and TdLN, while the spleen was unaffected. This indicated that FPC^2^‐IG‐IL‐2 was able to confer a localized effect within tumors. Furthermore, unlike the free IL‐2, which showed a burst release of IL‐2 after the intratumoral injection, FPC^2^‐IG‐IL‐2 injected tumors maintained IL‐2 levels after 10 days of injection.

High doses of IL‐2 have been required for clinical therapies due to its short half‐life, which introduces various toxicity‐related side effects. In order to reduce the IL‐2 dosage, several studies have attempted to increase the persistence of therapeutic IL‐2 in the bloodstream. One of these approaches was to attach polyethylene glycol to IL‐2 (PEGylation), which improved its in vivo retention by increasing the hydrodynamic size. Its clinical efficacy was promising when administered in combination with immune checkpoint inhibitor therapies.^34^, ^35^ Microencapsulation of IL‐2 with FPC^2^ allows a prolonged presence of IL‐2 within tumors, since IL‐2 is protected against proteases secreted by tumor cells and released in a controlled manner. The release mechanism of FPC^2^‐IG is initiated by the early dissolution of IG, followed by molecular diffusion and subsequent proteolysis of FPC^2^.

Another drawback of IL‐2 therapy is the expansion of Treg cells, which express high levels of IL‐2Rα. Low‐dose IL‐2 therapy primarily promotes Treg cell expansion, which has been used to treat individuals with autoimmune diseases, whereas high‐dose IL‐2 administration induces both Treg and CD8^+^T cell expansions.^36^ Our results indicated that IL‐2 released from FPC^2^‐IG showed an increased bioactivity toward CD8^+^T cells over Treg cells ex vivo. This is possibly due to the fucoidan‐bound released IL‐2 selectively binds to IL‐2Rβγ rather than IL‐2Rα. Since fucoidan can bind at the IL‐2Rα‐binding region of IL‐2, or the complex and bulky structure of fucoidan can cause a steric hindrance on IL‐2Rα.^37^ A similar mechanism has been reported in PEGylated IL‐2, which preferentially binds to IL‐2Rβγ by attaching PEG chains at the site where IL‐2 may bind to IL‐2Rα.^34^, ^38^ Indeed, treatments with PEGylated IL‐2, NKTR‐214 (developed by Nektar therapeutics) increased cytotoxic immune cell populations, including CD8^+^T and NK cells, with a limited effect on Treg cell expansion in both cancer patients and preclinical models.^39^, ^40^

FPC^2^‐IG‐IL‐2 also caused alterations to TIME. It increased the expansion of tumor‐infiltrating cytotoxic lymphocytes over Treg cells and reduced the frequencies of myeloid populations. Despite the immune‐favorable TME induced by FPC^2^‐IG‐IL‐2, a single injection of FPC^2^‐IG‐IL‐2 alone produced only a moderate antitumor efficacy in CT26‐bearing mice. This indicated that the increase of tumor‐infiltrating cytotoxic lymphocytes via the localized IL‐2 delivery of FPC^2^‐IG‐IL‐2 may not be effective enough to elicit antitumor immune responses compared to that of PEGylated IL‐2, which was reported to promote the proliferation and activation of T and NK cells not only within tumors but also within the peripheral blood.^34^ However, FPC^2^‐IG‐IL‐2 was fully capable of enhancing the antitumor efficacy of a PD‐1 blockade in CT26‐bearing mice. Furthermore, FPC^2^‐IG‐IL‐2 supported the adoptive transfer of tumor‐reactive CD8^+^T cells to increase their proliferative capacity and antitumor immune responses. Although IL‐2 is the key cytokine for maintaining T‐cell homeostasis, it can make differential effects on CD8^+^T cell differentiation and responses; depending on the strength and duration of its signaling.^27^, ^41^, ^42^ Strong IL‐2 signaling has been known to promote the generation of short‐lived terminal effector CD8^+^T cells, while low IL‐2 induces CD8^+^T cells to acquire a long‐lived memory phenotype; one of the key criteria for successful adoptive T‐cell therapy.^43^ We were able to observe that NY‐ESO‐1 TCR‐T cells adoptively transferred into A375 melanoma‐bearing NSG mice retained CD62L^+^stem cell memory and central memory populations when FPC^2^‐IG‐IL‐2 was administered. This implied that the sustained and controlled release of IL‐2 from FPC^2^‐IG provides ample stimulation for IL‐2 signaling. Considering the harsh in vivo conditions of NSG mice, where no endogenous IL‐2 is available for human T cells, it would be a reasonable assumption that FPC^2^‐IG‐IL‐2 allows NY‐ESO‐1 TCR‐T cells to survive and exert durable antitumor immune responses in immunosuppressive solid TME.

## 4 CONCLUSIONS

IL‐2 encapsulation by a marine‐inspired complex coacervate of fucoidan and PLL (FPC^2^) facilitates the sustained release and extended bioactivity of IL‐2 within tumors. The FPC^2^ delivery system surpasses the current strategies used for IL‐2 therapies by contributing to a reduction of toxicity and improvement in antitumor efficacy. Our findings systematically confirm that FPC^2^‐IG‐IL‐2 provides an immune favorable TME and promotes the quality of adoptively transferred tumor‐antigen‐specific T cells. Our approach thus represents a promising new treatment method for TCR‐engineered T‐cell therapies that target solid tumors.

## 5 MATERIALS AND METHODS

### 5.1 Zeta potential measurement

To determine the electric points for fucoidan from *Fucus Vesiculosus* (purity ≥95%, Sigma‐Aldrich) and poly‐l‐lysine (PLL; Molecular weight 15–30 kDa, Sigma‐Aldrich), the zeta potential was measured by a zeta potential analyzer (Malvern, Zetasizer Nano ZS90) at different pH values (4, 5.5, 7, 8.5, and 10.4) with constant polyelectrolyte concentration of 0.75 mg/ml. Polyelectrolytes were dissolved in different buffer solutions, and the buffers included 0.1 M sodium acetate (pH 4 and 5.5), 0.1 M Tris–HCl (pH 7 and 8.5), and 0.1 M sodium carbonate–bicarbonate (pH 10.4). The results from at least five samples were averaged to obtain each measurement.

### 5.2 Preparation of fucoidan/PLL complex coacervates (FPC^2^ )

Complex coacervation was achieved by mixing anionic fucoidan and cationic PLL. By varying the mixing ratio of fucoidan and PLL solutions, dissolved in Phosphate Buffered Saline (PBS; Gibco) at pH 7.4, the extent of coacervation was monitored by turbidity measurements.^21^ For turbidity tests, the stock solution of 3.15 mg/ml of each polyelectrolyte was prepared, and the total polyelectrolyte concentration was fixed at 3.15 mg/ml. A plate reader equipped with a UV–Vis spectrophotometer (Promega, GloMax®) was utilized at a wavelength of 600 nm for the turbidity measurements. All fucoidan/PLL complex coacervates (FPC^2^) were prepared immediately before turbidity measurements and measured at room temperature (RT). The results from triplicate samples were averaged to obtain each measurement. To observe the liquid micro‐droplets of FPC^2^, the mixture of Fucoidan and PLL solutions (wt/wt ratio of 7:3) was monitored by an optical microscope (Nikon, Eclipse TE2000‐U).^21^

### 5.3 In vitro cytocompatibility tests

To assess the cytocompatibility of FPC^2^, human dermal fibroblasts (HDFs) were seeded into a 96‐well plate (SPL) at a density of 10^3^ cells and were cultured in 100 μl of DMEM with high glucose (HyClone) supplemented with 10% fetal bovine serum (FBS; CellScience), 1% penicillin/streptomycin (Gibco), 1% l‐glutamine (Gibco), 1% MEM‐non‐essential amino acid (Gibco), 55 μM 2‐mercaptoethanol (Gibco) and 1 mM sodium pyruvate (Gibco). After incubation for 24 h, 50 μl of FPC^2^ suspension was treated to cells at four different concentrations: 12.5, 6.25, 3.13, and 1.56 mg/ml. HDFs were then incubated for another 24 and 48 h at 37°C and 5% CO_2_. Cell viability was quantitatively determined using the Cell Counting Kit‐8 assay (CCK‐8; Dojindo) with blank medium and 15% (v/v) dimethyl sulfoxide (DMSO) as negative and positive controls, respectively. The results from at least triplicate samples were averaged to obtain each measurement.

To examine the cytoskeletal structure of HDFs cultured with FPC^2^, the cells were fixed in 0.5% paraformaldehyde in preheated PBS for 30 min at 37°C, after incubation for 24 h. For cell permeabilization, 0.1% Triton X‐100 (Sigma‐Aldrich) in PBS was added to fixed cells for 5 min. The cells were then immersed in Alexa Fluor 594‐phalloidin solution (Thermo Fisher, 1:200 dilution) for 30 min. The nuclei were counterstained with 0.1 μg/ml DAPI (Invitrogen) for 5 min at RT. For visualization of FPC^2^, fluorescein‐labeled bovine serum albumin (BSA‐FITC; Sigma‐Aldrich)‐laden FPC^2^ was prepared by adding the BSA‐FITC/PLL mixture into the fucoidan solution. The fluorescence‐stained cells were visualized under a fluorescence microscope (Nikon, Eclipse TE2000‐U). A tissue culture plate (TCP) was used as a positive control.

### 5.4 Preparation of fucoidan/PLL coacervates‐laden injectable hydrogel (FPC^2^‐IG)

Fucoidan/PLL complex coacervates‐laden injectable hydrogel (FPC^2^‐IG) was prepared by mixing 25 μl of 6.25 mg/ml FPC^2^ suspension, and 225 μl of 16.7% (w/v) of Pluronic® F‐127 solution in PBS containing sodium bicarbonate (NaHCO_3_) at different concentrations of 50, 100, and 200 mM on ice. To evaluate the effect of NaHCO_3_ concentration on the size of FPC^2^ liquid micro‐droplets, FPC^2^‐IG solution was incubated at 4°C for 48 h and then observed by an optical microscope. To calculate the average diameter of liquid droplets, at least 1000 liquid droplets were analyzed using Image J software (National Institute of Health).

### 5.5 Protein encapsulation into FPC^2^

To confirm protein encapsulation into FPC^2^, BSA‐FITC and human interleukin‐2 (IL‐2; Peprotech) were used as a model protein and a key cytokine for improved anti‐tumor immune response, respectively. Protein‐laden FPC^2^ was prepared using two different mixing orders: (Method 1) addition of protein solution to fucoidan solution to form protein–fucoidan intermediate complex, followed by adding the mixture into PLL solution with a fucoidan:PLL weight ratio of 7:3 and 5:5; (Method 2) addition of protein solution to PLL solution to form protein–PLL intermediate complex, followed by adding the mixture into fucoidan solution with a fucoidan:PLL weight ratio of 7:3 and 5:5. The total concentration of fucoidan and PLL was fixed at 6.25 mg/ml, and the final concentration of BSA‐FITC and IL‐2 was 50 μg/ml and 25 μg/ml, respectively. To determine protein encapsulation yield, FPC^2^‐BSA and FPC^2^‐IL‐2 samples were centrifuged for 10 min at 12,000 *g*.^44^ After centrifugation, the supernatant was collected using a micropipette and the coacervate phase was left in the bottom of centrifuge tubes. Using the supernatant, the amount of the unloaded BSA‐FITC and IL‐2 was analyzed by a fluorescence spectroscopy with λ_ex_ = 488 nm and λ_em_ = 522 nm (Promega, GloMax®) and human IL‐2 ELISA kit (Peprotech) according to the manufacturer's protocol, respectively.

At various protein concentrations, the encapsulation capacity of FPC^2^ was also examined. For BSA encapsulation, FPC^2^‐BSA with different BSA‐FITC concentrations (12.5, 25, 50, 100, and 200 μg/ml) were prepared using Method 1 at a fucoidan:PLL weight ratio of 5:5. For IL‐2 encapsulation, FPC^2^‐IL‐2 with different IL‐2 concentrations (12.5, 25, 50, 100, 200, and 600 μg/ml) were prepared using Method 2 at a fucoidan:PLL weight ratio of 7:3. The protein encapsulation yield was quantified as described above. The results from five samples were averaged to obtain each measurement.

### 5.6 In vitro protein release from FPC^2^‐IG

For the preparation of BSA or IL‐2‐loaded FPC^2^‐IG, 10 μl of protein‐loaded FPC^2^ suspension was formed as described above and mixed with 90 μl of 16.7% (w/v) of Pluronic® F‐127 solution in PBS containing 100 mM NaHCO_3_ on ice. The final concentration of BSA‐FITC and IL‐2 were 25 and 60 μg/ml, respectively. After thermo‐gelation at 37°C for 1 h, FPC^2^‐IG samples were submerged in 100 μl of preheated PBS and incubated at 37°C. At predetermined time points, FPC^2^‐IG samples were centrifuged at 12,000 *g* and 37°C for 3 min. Hundred microliters of the supernatant was collected and stored at −80°C. Hundred microliters of fresh PBS was then added to FPC^2^‐IG. The concentrations of BSA and IL‐2 in the releasate were determined by a fluorescence spectroscopy and IL‐2 ELISA kit, respectively. The results from triplicate samples were averaged to obtain each measurement.

### 5.7 In vivo protein retention test

For in vivo tracking of the protein loaded in FPC^2^‐IG, BSA (Sigma‐Aldrich) was labeled with a near‐infrared fluorophore containing an NHS ester (Vivotag 680XL, PerkinElmer) according to the manufacturer's protocol. Briefly, BSA was dissolved in 0.1 M sodium carbonate/bicarbonate buffer (pH 8.4) at 1 mg/ml and mixed with 10 times molar excess of Vivotag 680XL. The mixture was incubated in the dark at RT for 4 h while shaking. Excess of nonreacted fluorophore was removed using a centrifugal filter with a 3 kDa molecular weight cutoff (Amicon® Ultra‐0.5 ml, MilliporeSigma). The labeled BSA was loaded into FPC^2^‐IG as described above, and the final concentration of BSA‐Vivotag 680XL was 25 μg/ml.

All surgical procedures were conducted according to the Korea Institute of Science and Technology (KIST) Institutional Animal Care and Use committee protocols (KIST‐2020‐082). For in vivo BSA tracking studies, 60 μl of FPC^2^‐IG‐BSA was subcutaneously injected into backs of 8‐week‐old male BALB/c nude mouse (DBL) via insulin syringes (28.5 gauge, 0.5‐inch needles; BD). FPC^2^‐BSA and PBS containing BSA‐Vivotag 680XL were also injected as control groups. In vivo fluorescence imaging (λ_ex_ = 675 nm and λ_em_ = 720 nm) was performed using an IVIS Spectrum platform (PerkinElmer). Longitudinal in vivo imaging was obtained following subcutaneous injection on day 0 (4 h) as well as on day 1, 4, 7, 15, and 21. Average radiant efficiency was evaluated using a 6.74‐cm^2^ elliptical region of interest in Living Image Software (PerkinElmer), and the fluorescent signal was normalized to day 0 values. The results from triplicate samples were averaged to obtain each measurement.

### 5.8 Evaluation of the encapsulated protein from proteolysis

To verify the protective effects of FPC^2^ for the encapsulated protein from proteolysis, FPC^2^‐BSA was loaded into collagen hydrogels, and FPC^2^‐collagen gel composites were treated with collagenase solution. Briefly, BSA‐FITC was encapsulated in FPC^2^ as previously described, followed by incubation with collagen prehydrogel solution containing collagen type I solution from rat tail (Corning), 10× PBS, 1 N NaOH (Thermo Fisher), and distilled water (DW) on ice. The final concentrations of BSA‐FITC and collagen gel were 100 μg/ml and 3 mg/ml. As a control group, BSA‐FITC was mixed with collagen prehydrogel solution without coacervation. For gelation, the collagen prehydrogel solutions containing BSA‐FITC were incubated at 37°C for 1 h. Hundred microliters of 29 U/ml collagenase II in PBS was then added to 100 μl of the collagen gel samples, and further incubated at 37°C. At predetermined time points (1, 3, and 16 h), samples were centrifuged at 12,000 *g* and 37°C for 3 min. Hundred microliters of the supernatant was collected for visual inspection and fluorescence quantification using a microscope and fluorescence spectroscope, respectively. The levels of remaining BSA‐FITC (protected BSA) were quantified by subtracting the detected fluorescence in the supernatant (BSA exposed to enzyme) from the total fluorescence of initial BSA‐FITC. The results from four samples were averaged to obtain each measurement.

### 5.9 Human T‐cell proliferation assay

Cryopreserved human peripheral blood mononuclear cells (PBMC) were purchased from AllCells (Emeryvill, CA). Total CD3^+^T cells were isolated by negative selection with a magnetic isolation kit and CD4^+^T, CD8^+^T, and regulatory T (Treg) cells were then sorted by flow cytometry using an MA900 cell sorter (SONY). Sorted T cells were stained with CellTrace Violet (CTV; Invitrogen) and stimulated with Dynabeads™ Human T‐Activator CD3/CD28 (Invitrogen) at a cell‐to‐bead ratio of 2:1 in complete RPMI 1640 medium with unencapsulated IL‐2 (100 ng/ml; Peprotech) or encapsulated IL‐2 (FPC^2^‐IL‐2) for 5 days. T‐cell proliferation was measured by assessing CTV dilution by flow cytometry. The proliferation index was calculated by FlowJo software (v.10.8.0, Tree Star).

### 5.10 Cell lines

CT26 colon carcinoma (CRL‐2638), A375 melanoma (CRL‐1619), and B16‐F10 (CRL‐6475) melanoma were obtained from American Type Culture Collection (ATCC). 293FT cells were purchased from Invitrogen (Carlsbad, CA). B16‐OVA cells were generated using Lipofectamine 3000 (Invitrogen, Waltham, MA) with a cDNA encoding ovalbumin (OVA). Single colonies were isolated, expanded, and characterized for OVA expression. In order to generate A375 cells stably expressing a firefly luciferase (A375‐LUC), A375 cells were transfected with a plasmid pSBbi‐BP (Addgene plasmid #60512) expressing firefly luciferase gene and selected using puromycin.^45^ All tumor cell lines and genetically modified lines derived from them were cultured in RPMI‐1640 or DMEM medium with 10% heated‐inactivated FBS and 1× penicillin/streptomycin/glutamine (Thermo Fisher Scientific, San Francisco, CA). All cell lines were tested for mycoplasma contamination by qPCR analysis (BioMax, South Korea).^46^

### 5.11 Generation of human NY‐ESO‐1 TCR‐T cells

A lentiviral vector encoding a TCR that recognizes the HLA‐A2 restricted NY‐ESO‐1 peptide antigen (NY‐ESO‐1:157‐165) was generated in the pHR lentiviral backbone (Addgene plasmid #79125). Lentivirus was generated via the transient transfection of 293FT cells with the packaging plasmids psPAX2 (Addgene plasmid #12260) and pVSVg (Addgene plasmid #8454). Human primary CD8^+^T cells were stimulated with a Dynabeads Human T‐Activator CD3/CD28 (Invitrogen) in the presence of recombinant human IL‐2 (300 IU/ml) and transduced with a lentivirus encoding a NY‐ESO‐1 TCR by spinoculation in the presence of polybrene (8 μg/ml). The expression of NY‐ESO‐1 TCR was measured by flow cytometric analysis using an anti‐human Vβ13.1 TCR chain antibody (BioLegend).

### 5.12 Murine tumor models

Mouse tumor experiments were approved and supervised by the Asan Medical Center Institutional Animal Care and Use Committee (202012288), in accordance with AALAC guidelines. C57BL/6, BALB/c, and NOD‐SCID IL2Rγnull (NSG) mice of 6–8 weeks of age were obtained from JA Bio (Suwon, Korea). OT‐I T‐cell receptor (TCR)–transgenic mice [C57BL/6‐Tg(TcraTcrb)1100Mjb/J] and CD45.1 [B6.SJL‐PtprcaPepcb/BoyJ] mice were purchased from The Jackson Laboratory. CD45.1^+^ OT‐I mice were obtained by crossbreeding. All mice were bred and maintained in pathogen‐free conditions in the animal facility of Asan Medical Center. Mouse tumor cells were cultured and passaged twice before inoculation, ready for tumor challenge testing. CT26 cells were resuspended in Hank's balanced salt solution (HBSS) without phenol red (Welgene, Korea) and subcutaneously injected into the left flank of BALB/c mice. CD8^+^ T‐cell responses to CT26 antigen AH1 (SPSYVYHQF) were assessed using the production of IFN‐γ following peptide stimulation. For adoptive transfer experiments, C57BL/6 mice were subcutaneously injected with 2 × 10^5^ B16‐OVA tumor cells. CD8^+^ T cells were isolated from CD45.1^+^ OT‐I mice by negative selection using a CD8 isolation kit (STEMCELL Technologies), activated with SIINFEKL peptide (OVA257‐264), and human IL‐2. Naïve CD8^+^ T cells were purified with an EasySep™ Mouse Naïve CD8^+^ T Cell Isolation Kit (STEMCELL Technologies). When the tumors reached a volume of approximately 150 mm^3^, 50 μl of FPC^2^‐IG or FPC^2^‐IG‐IL2 was intratumorally administered via an insulin syringe. One day later, mice received 1 million peptide‐activated OT‐I T cells intravenously. For human NY‐ESO TCR‐T experiments, A375 cells were subcutaneously implanted into the flank of NSG mice. These mice were then randomly grouped when tumor size reached approximately 100–150 mm^3^ and intratumorally injected with FPC^2^‐IG or FPC^2^‐IG‐IL2. One day after these injections, 1 million NY‐ESO‐1 TCR‐T cells were intravenously transferred into the tumor‐bearing mice. Tumor growth was monitored with a caliper measurement twice weekly.

### 5.13 Measurement of the IL‐2 concentration within tumors

Tumor tissues were frozen in dry ice and processed in 1× PBS containing 0.1% BSA and protease inhibitor cocktail (MilliporeSigma) by homogenization using an IKA ULTRA‐TURRAX T‐25 (IKA, Germany). Tumor homogenates were centrifuged, and human IL‐2 in the supernatant was analyzed with an ELISA kit (BioLegend) according to the manufacturer's instructions.

### 5.14 TIL analysis

Mouse tumors were dissected and digested with collagenase IV (250 unit/ml, Worthington Biochemical Cooperation) and DNase I (100 μg/ml, Roche Diagnostics GmbH) in HBSS using a gentleMACS dissociator (Miltenyi Biotec). TILs were enriched by Ficoll–Hypaque (MilliporeSigma) gradient centrifugation and single cells were recovered. Finally, the cell viability was determined by trypan blue exclusion.

### 5.15 Antibodies and flow cytometry

Human and mouse single cells were blocked with human TruStain FcX™ (BioLegend) and anti‐mouse CD16/32 (BioLegend), respectively, and then stained with the indicated antibodies in fluorescence‐activated cell sorter (FACS) buffer (PBS containing 2% FBS and 0.1% sodium azide) for 20 min at 4°C. Dead cells were excluded using Zombie NIR Fixable viability dye (BioLegend). For intracellular staining, cells were stained with antibodies to surface markers and then fixed and permeabilized with Cyto‐Fast Fix‐Perm buffer (BioLegend), followed by staining with indicated antibodies diluted in Cyto‐Fast™ Perm wash buffer (BioLegend). The following fluorescent dye‐conjugated anti‐human antibodies were used: anti‐CD3 (HIT3a), anti‐CD4 (OKT4), anti‐CD8 (RPA‐T8), anti‐CD127 (A019D5), anti‐CD62L (DREG‐56), anti‐CD45RO (UCHL1), anti‐TIGIT (A15153G), anti‐PD‐1 (EH12.2H7), anti‐Lag3 (11C3C65), anti‐CD25 (BC96), anti‐Foxp3 (259D), anti‐CCR8(L263G8), anti‐GITR (108–17), and anti‐OX40 (Ber‐ACT35). Antibodies against mouse proteins were as follows: anti‐CD45 (30‐F11), anti‐CD45.1 (A20), anti‐CD45.2 (104), anti‐CD3 (17A2), anti‐CD4 (RM4‐5), anti‐CD8 (53–6.7), anti‐NK1.1 (PK136), anti‐CD11b (M1/70), anti‐CD11c (N418), anti‐I‐A^b^ (AF6‐120.1), anti‐CD19 (6D5), anti‐PD‐1 (29F.1A12), anti‐Tim3 (B8.2C12), anti‐IFN‐γ (XMG1.2), and anti‐Ki‐67 (11F6) (all purchased from BioLegend). The acquisition of FACS data was performed on a CytoFLEX flow cytometer (Beckman Coulter, Brea, CA) and analyzed with FlowJo software (v.10.5.3, TreeStar). The automated analysis of multi‐parameter flow cytometry data was subjected to the *t*‐SNE (*t*‐Distribution Stochastic Neighbor Embedding)‐CUDA algorithm provided by Cytobank software (Beckman Coulter, Brea, CA). An equal number of cells (50,000 cells) from each FACS file were used to generate *t*‐SNE plots.

### 5.16 In situ immunofluorescence staining of TILs

CT26 tumors were immediately fixed in 4% paraformaldehyde for 3 h at 4°C and incubated in 30% sucrose overnight at 4°C, followed by optimal cutting temperature compound (OCT, Tissue‐Tek). Frozen sections were cut using a microtome‐cryostat (Leica CM1860) and stored at −20°C. Slide‐mounted tissues were blocked with 1x TBS containing 2% BSA and 0.1% Triton X‐100. The slides were incubated with a primary antibody directed against CD8 antigen (BioLegend; 100701, 1:100) followed by a secondary antibody coupled to Alexa Fluor 488 (BioLegend; 405418, 1:50). Tumor sections were counterstained with DAPI (BioLegend; 422801, 1:1000) and mounted in an aqueous mounting medium (Biomeda; M01). Imaging of the fluorescence of the stained samples was performed with a confocal microscope LSM880 (Carl Zeiss Microscopy).

### 5.17 Statistical analysis

Statistical analyses were conducted using GraphPad Prism 8.0. Statistical significance was determined with the two‐tailed paired or unpaired Student *t*‐test, or two‐way ANOVA with Tukey's multiple comparisons. Values of *p* < 0.05 were considered statistically significant (*p < 0.05, **p < 0.01, ***p < 0.001, and ****p < 0.0001). Details of the statistical tests used are provided in the individual figure legends.

## AUTHOR CONTRIBUTIONS

**Eun Young Jeon:** Conceptualization (equal); data curation (equal); formal analysis (equal); investigation (equal); methodology (equal); visualization (equal); writing – original draft (equal); writing – review and editing (equal). **Da‐som Choi:** Data curation (equal); investigation (equal). **Seunghyun Choi:** Investigation (supporting). **Ju‐young Won:** Investigation (supporting). **Yunju Jo:** Investigation (supporting). **Hye‐bin Kim:** Investigation (supporting). **Youngmee Jung:** Project administration (supporting); resources (supporting). **Sang Chul Shin:** Investigation (supporting). **Hophil Min:** Investigation (supporting). **Hae Woong Choi:** Project administration (supporting); resources (supporting). **Myeong Sup Lee:** Project administration (supporting); resources (supporting). **Yoon Park:** Conceptualization (equal); data curation (equal); formal analysis (equal); funding acquisition (equal); methodology (equal); project administration (equal); supervision (lead); writing – original draft (lead); writing – review and editing (lead). **Justin J. Chung:** Conceptualization (equal); funding acquisition (equal); project administration (equal); supervision (lead); writing – original draft (lead); writing – review and editing (lead). **hyung‐seung jin:** Conceptualization (equal); data curation (equal); formal analysis (equal); funding acquisition (equal); methodology (equal); project administration (equal); supervision (lead); writing – original draft (lead); writing – review and editing (lead).

## CONFLICT OF INTEREST

The authors declare that they have no known competing financial interests or personal relationships that could have appeared to influence the work reported in this article.

### PEER REVIEW

The peer review history for this article is available at https://publons.com/publon/10.1002/btm2.10362.

## Supporting information


**Appendix S1** Supporting Information

## Data Availability

The data that support the findings of this study are available from the corresponding author upon reasonable request.

## References

[1] Morgan RA , Dudley ME , Wunderlich JR , et al. Cancer regression in patients after transfer of genetically engineered lymphocytes. Science. 2006;314(5796):126‐129. 16946036 10.1126/science.1129003 PMC2267026

[2] Rosenberg SA , Restifo NP . Adoptive cell transfer as personalized immunotherapy for human cancer. Science. 2015;348(6230):62‐68. 25838374 10.1126/science.aaa4967 PMC6295668

[3] Zhao L , Cao YJ . Engineered T cell therapy for cancer in the clinic. Front Immunol. 2019;10:2250. 31681259 10.3389/fimmu.2019.02250 PMC6798078

[4] Maude SL , Laetsch TW , Buechner J , et al. Tisagenlecleucel in children and young adults with B‐cell lymphoblastic leukemia. N Engl J Med. 2018;378(5):439‐448. 29385370 10.1056/NEJMoa1709866 PMC5996391

[5] Robbins PF , Morgan RA , Feldman SA , et al. Tumor regression in patients with metastatic synovial cell sarcoma and melanoma using genetically engineered lymphocytes reactive with NY‐ESO‐1. J Clin Oncol. 2011;29(7):917‐924. 21282551 10.1200/JCO.2010.32.2537 PMC3068063

[6] Robbins PF , Kassim SH , Tran TL , et al. A pilot trial using lymphocytes genetically engineered with an NY‐ESO‐1‐reactive T‐cell receptor: long‐term follow‐up and correlates with response. Clin Cancer Res. 2015;21(5):1019‐1027. 25538264 10.1158/1078-0432.CCR-14-2708 PMC4361810

[7] Rodriguez‐Garcia A , Palazon A , Noguera‐Ortega E , Powell DJ Jr , Guedan S . CAR‐T cells hit the tumor microenvironment: strategies to overcome tumor escape. Front Immunol. 2020;11:1109. 32625204 10.3389/fimmu.2020.01109 PMC7311654

[8] Lee S , Margolin K . Cytokines in cancer immunotherapy. Cancers (Basel). 2011;3(4):3856‐3893. 24213115 10.3390/cancers3043856 PMC3763400

[9] Smyth MJ , Cretney E , Kershaw MH , Hayakawa Y . Cytokines in cancer immunity and immunotherapy. Immunol Rev. 2004;202:275‐293. 15546400 10.1111/j.0105-2896.2004.00199.x

[10] Rosenberg SA . IL‐2: the first effective immunotherapy for human cancer. J Immunol. 2014;192(12):5451‐5458. 24907378 10.4049/jimmunol.1490019 PMC6293462

[11] Atkins MB , Lotze MT , Dutcher JP , et al. High‐dose recombinant interleukin 2 therapy for patients with metastatic melanoma: analysis of 270 patients treated between 1985 and 1993. J Clin Oncol. 1999;17(7):2105‐2116. 10561265 10.1200/JCO.1999.17.7.2105

[12] Zhao Z , Zheng L , Chen W , Weng W , Song J , Ji J . Delivery strategies of cancer immunotherapy: recent advances and future perspectives. J Hematol Oncol. 2019;12(1):126. 31779642 10.1186/s13045-019-0817-3 PMC6883629

[13] Gandhi NS , Mancera RL . The structure of glycosaminoglycans and their interactions with proteins. Chem Biol Drug Des. 2008;72(6):455‐482. 19090915 10.1111/j.1747-0285.2008.00741.x

[14] Miller T , Goude MC , McDevitt TC , Temenoff JS . Molecular engineering of glycosaminoglycan chemistry for biomolecule delivery. Acta Biomater. 2014;10(4):1705‐1719. 24121191 10.1016/j.actbio.2013.09.039 PMC3960340

[15] McCaffrey TA , Falcone DJ , Vicente D , Du B , Consigli S , Borth W . Protection of transforming growth factor‐beta 1 activity by heparin and fucoidan. J Cell Physiol. 1994;159(1):51‐59. 7511146 10.1002/jcp.1041590108

[16] Mummery RS , Rider CC . Characterization of the heparin‐binding properties of IL‐6. J Immunol. 2000;165(10):5671‐5679. 11067924 10.4049/jimmunol.165.10.5671

[17] Luthuli S , Wu S , Cheng Y , Zheng X , Wu M , Tong H . Therapeutic effects of Fucoidan: a review on recent studies. Mar Drugs. 2019;17(9):487. 31438588 10.3390/md17090487 PMC6780838

[18] Atashrazm F , Lowenthal RM , Woods GM , Holloway AF , Dickinson JL . Fucoidan and cancer: a multifunctional molecule with anti‐tumor potential. Mar Drugs. 2015;13(4):2327‐2346. 25874926 10.3390/md13042327 PMC4413214

[19] Wei W , Tan Y , Martinez Rodriguez NR , Yu J , Israelachvili JN , Waite JH . A mussel‐derived one component adhesive coacervate. Acta Biomater. 2014;10(4):1663‐1670. 24060881 10.1016/j.actbio.2013.09.007 PMC3960351

[20] Zhao H , Sun C , Stewart RJ , Waite JH . Cement proteins of the tube‐building polychaete *Phragmatopoma californica* . J Biol Chem. 2005;280(52):42938‐42944. 16227622 10.1074/jbc.M508457200

[21] Priftis D , Tirrell M . Phase behaviour and complex coacervation of aqueous polypeptide solutions. Soft Matter. 2012;8(36):9396‐9405.

[22] Huang KY , Yoo HY , Jho Y , Han S , Hwang DS . Bicontinuous fluid structure with low cohesive energy: molecular basis for exceptionally low interfacial tension of complex Coacervate fluids. ACS Nano. 2016;10(5):5051‐5062. 27152954 10.1021/acsnano.5b07787

[23] Gouin S . Microencapsulation: industrial appraisal of existing technologies and trends. Trends Food Sci Technol. 2004;15(7–8):330‐347.

[24] Jin HS , Choi DS , Ko M , et al. Extracellular pH modulating injectable gel for enhancing immune checkpoint inhibitor therapy. J Control Release. 2019;315:65‐75. 31669264 10.1016/j.jconrel.2019.10.041

[25] Moore T , Croy S , Mallapragada S , Pandit N . Experimental investigation and mathematical modeling of Pluronic F127 gel dissolution: drug release in stirred systems. J Control Release. 2000;67(2–3):191‐202. 10825553 10.1016/s0168-3659(00)00215-7

[26] Kessenbrock K , Plaks V , Werb Z . Matrix metalloproteinases: regulators of the tumor microenvironment. Cell. 2010;141(1):52‐67. 20371345 10.1016/j.cell.2010.03.015 PMC2862057

[27] Pipkin ME , Sacks JA , Cruz‐Guilloty F , Lichtenheld MG , Bevan MJ , Rao A . Interleukin‐2 and inflammation induce distinct transcriptional programs that promote the differentiation of effector cytolytic T cells. Immunity. 2010;32(1):79‐90. 20096607 10.1016/j.immuni.2009.11.012 PMC2906224

[28] Ohue Y , Nishikawa H . Regulatory T (Treg) cells in cancer: can Treg cells be a new therapeutic target? Cancer Sci. 2019;110(7):2080‐2089. 31102428 10.1111/cas.14069 PMC6609813

[29] Kim HJ , Hwang BH , Lim S , Choi BH , Kang SH , Cha HJ . Mussel adhesion‐employed water‐immiscible fluid bioadhesive for urinary fistula sealing. Biomaterials. 2015;72:104‐111. 26352517 10.1016/j.biomaterials.2015.08.055

[30] Park TY , Jeon EY , Kim HJ , Choi BH , Cha HJ . Prolonged cell persistence with enhanced multipotency and rapid angiogenesis of hypoxia pre‐conditioned stem cells encapsulated in marine‐inspired adhesive and immiscible liquid micro‐droplets. Acta Biomater. 2019;86:257‐268. 30639576 10.1016/j.actbio.2019.01.007

[31] Shukla SC , Singh A , Pandey AK , Mishra A . Review on production and medical applications of epsilon‐polylysine. Biochem Eng J. 2012;65:70‐81.

[32] Hachim D , Whittaker TE , Kim H , Stevens MM . Glycosaminoglycan‐based biomaterials for growth factor and cytokine delivery: making the right choices. J Control Release. 2019;313:131‐147. 31629041 10.1016/j.jconrel.2019.10.018 PMC6900262

[33] Mortara L , Balza E , Bruno A , Poggi A , Orecchia P , Carnemolla B . Anti‐cancer therapies employing IL‐2 cytokine tumor targeting: contribution of innate, adaptive and immunosuppressive cells in the anti‐tumor efficacy. Front Immunol. 2018;9:2905. 30619269 10.3389/fimmu.2018.02905 PMC6305397

[34] Charych DH , Hoch U , Langowski JL , et al. NKTR‐214, an engineered cytokine with biased IL2 receptor binding, increased tumor exposure, and marked efficacy in mouse tumor models. Clin Cancer Res. 2016;22(3):680‐690. 26832745 10.1158/1078-0432.CCR-15-1631

[35] Maier KE , Rusconi CP , Levy M . To PEGylate or not to PEGylate therapeutics? Cell Chem Biol. 2019;26(5):615‐616. 31100258 10.1016/j.chembiol.2019.04.014

[36] Jiang T , Zhou C , Ren S . Role of IL‐2 in cancer immunotherapy. Onco Targets Ther. 2016;5(6):e1163462. 10.1080/2162402X.2016.1163462 PMC4938354 27471638

[37] Li B , Lu F , Wei X , Zhao R . Fucoidan: structure and bioactivity. Molecules. 2008;13(8):1671‐1695. 18794778 10.3390/molecules13081671 PMC6245444

[38] Charych D , Khalili S , Dixit V , et al. Modeling the receptor pharmacology, pharmacokinetics, and pharmacodynamics of NKTR‐214, a kinetically‐controlled interleukin‐2 (IL2) receptor agonist for cancer immunotherapy. PLoS One. 2017;12(7):e0179431. 28678791 10.1371/journal.pone.0179431 PMC5497954

[39] Bentebibel SE , Hurwitz ME , Bernatchez C , et al. A first‐in‐human study and biomarker analysis of NKTR‐214, a novel IL2Rbetagamma‐biased cytokine, in patients with advanced or metastatic solid tumors. Cancer Discov. 2019;9(6):711‐721. 30988166 10.1158/2159-8290.CD-18-1495

[40] Parisi G , Saco JD , Salazar FB , et al. Persistence of adoptively transferred T cells with a kinetically engineered IL‐2 receptor agonist. Nat Commun. 2020;11(1):660. 32005809 10.1038/s41467-019-12901-3 PMC6994533

[41] Kalia V , Sarkar S , Subramaniam S , Haining WN , Smith KA , Ahmed R . Prolonged interleukin‐2Ralpha expression on virus‐specific CD8+ T cells favors terminal‐effector differentiation in vivo. Immunity. 2010;32(1):91‐103. 20096608 10.1016/j.immuni.2009.11.010

[42] Kalia V , Sarkar S . Regulation of effector and memory CD8 T cell differentiation by IL‐2‐a balancing act. Front Immunol. 2018;9:2987. 30619342 10.3389/fimmu.2018.02987 PMC6306427

[43] Restifo NP , Dudley ME , Rosenberg SA . Adoptive immunotherapy for cancer: harnessing the T cell response. Nat Rev Immunol. 2012;12(4):269‐281. 22437939 10.1038/nri3191 PMC6292222

[44] Black KA , Priftis D , Perry SL , Yip J , Byun WY , Tirrell M . Protein encapsulation via polypeptide complex Coacervation. ACS Macro Lett. 2014;3(10):1088‐1091. 35610798 10.1021/mz500529v

[45] Jin HS , Ko M , Choi DS , et al. CD226(hi)CD8(+) T cells are a prerequisite for anti‐TIGIT immunotherapy. Cancer Immunol Res. 2020;8(7):912‐925. 32265229 10.1158/2326-6066.CIR-19-0877

[46] Li TW , Fu JX , Zeng ZX , et al. TIMER2.0 for analysis of tumor‐infiltrating immune cells. Nucleic Acids Res. 2020;48(W1):W509‐W514. 32442275 10.1093/nar/gkaa407 PMC7319575

