# Supplementary material for: Enhancing adoptive T‐cell therapy with fucoidan‐based IL‐2 delivery microcapsules
Source: Bioeng Transl Med. 2022 Jul 5;8(1):e10362. doi: 10.1002/btm2.10362 (PMC9842027; doi:10.1002/btm2.10362)
Supplement: Supplementary file 1 — Appendix S1 Supporting Information [file BTM2-8-e10362-s001.pdf]

Supplementary data

Enhancing adoptive T cell therapy with fucoidan-based IL-2 delivery microcapsules

Eun Young Jeon, Da-som Choi, Seunghyun Choi, Ju-young Won, Yunju Jo, Hye-bin Kim, Youngmee Jung, Sang Chul Shin, Hophil Min, Hae Woong Choi, Myeong Sup Lee, Yoon Park*, Justin J. Chung*, and Hyung-seung Jin*

Supplemental Figures

Supplementary Fig. 1. Underwater stability of bulk FPC^2. To assess the water-immiscibility and underwater stability of FPC^2, the letter 'KIST' was written in a petri-dish containing PBS using bulk FPC^2 through a 22G syringe and monitored for 2 months. Bulk FPC^2 was prepared via the centrifugation of an FPC^2 suspension.

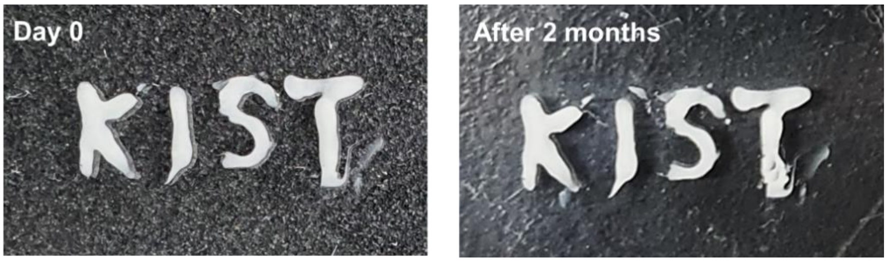

Supplementary Fig. 2. Protein protection effects of FPC^2 against proteolysis. Optical microscopic (left) and fluorescent images (right) of the lysates of (a) free BSA-laden collagen gels and (c) FPC^2-BSA-laden collagen gels after collagenase treatment. Scale bar = 100 μm. Visualization of (b) the dissolution of BSA-FITC from fully degraded collagen gels and (d) the protective effects of FPC^2 for the encapsulated BSA against proteolysis.

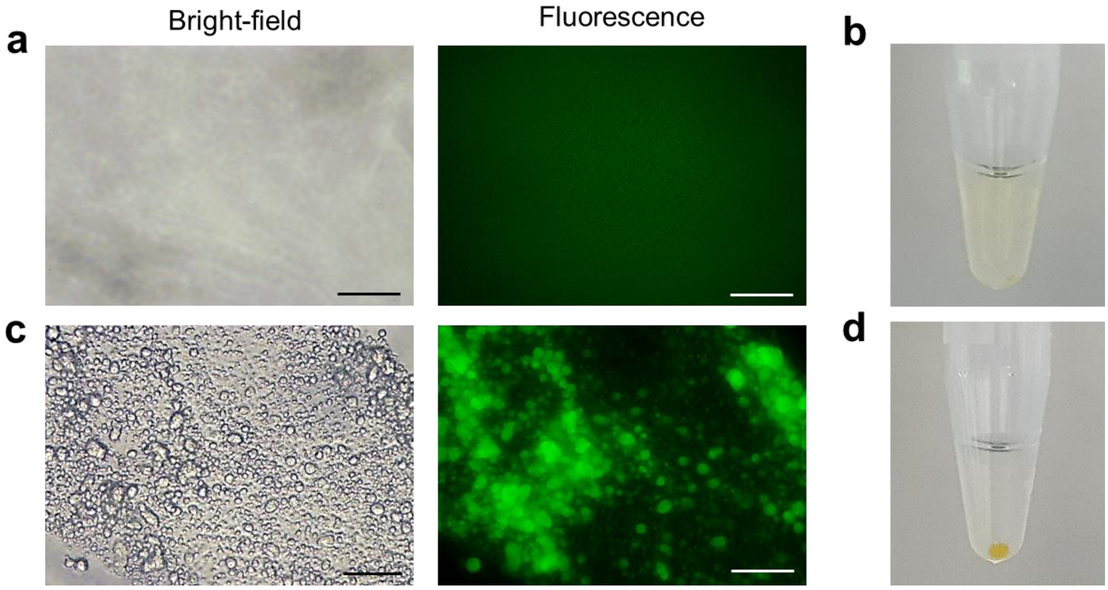

Supplementary Fig. 3. Schematic illustrations of IL-2 encapsulation into FPC[2] using two different mixing orders.

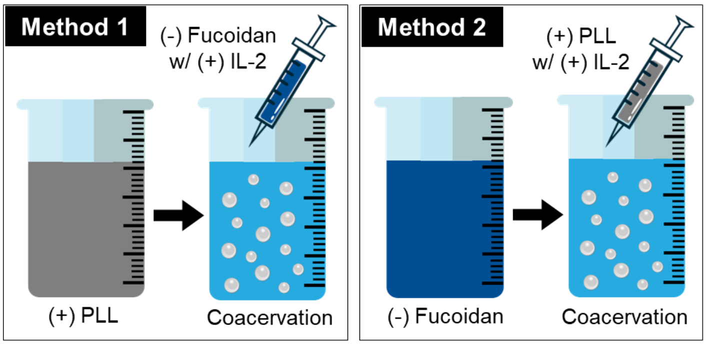

Supplementary Fig. 4. Effects of FPC^2-IG-IL-2 on the recruitment of CD8+ T cells to the TME.

(a) Treatment schedule for the analysis of CD8$^+$ T cells in TME.

(b) Immunofluorescence staining showing CD8$^+$ T cells in tumor sections on day 7 post treatment. Scale bar = 100 μm. The population of CD8$^+$ T cells in the tumor treated with a single dose of FPC^2-IG-IL-2 was increased compared to other groups.

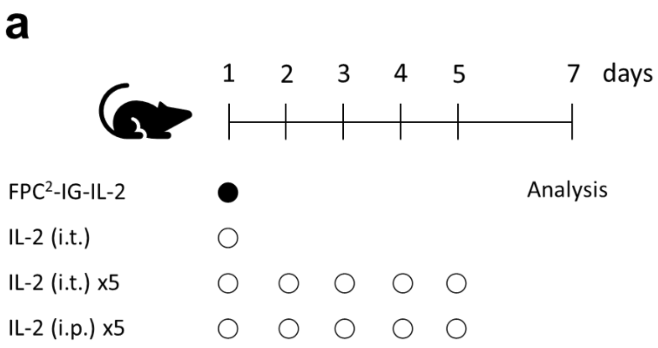

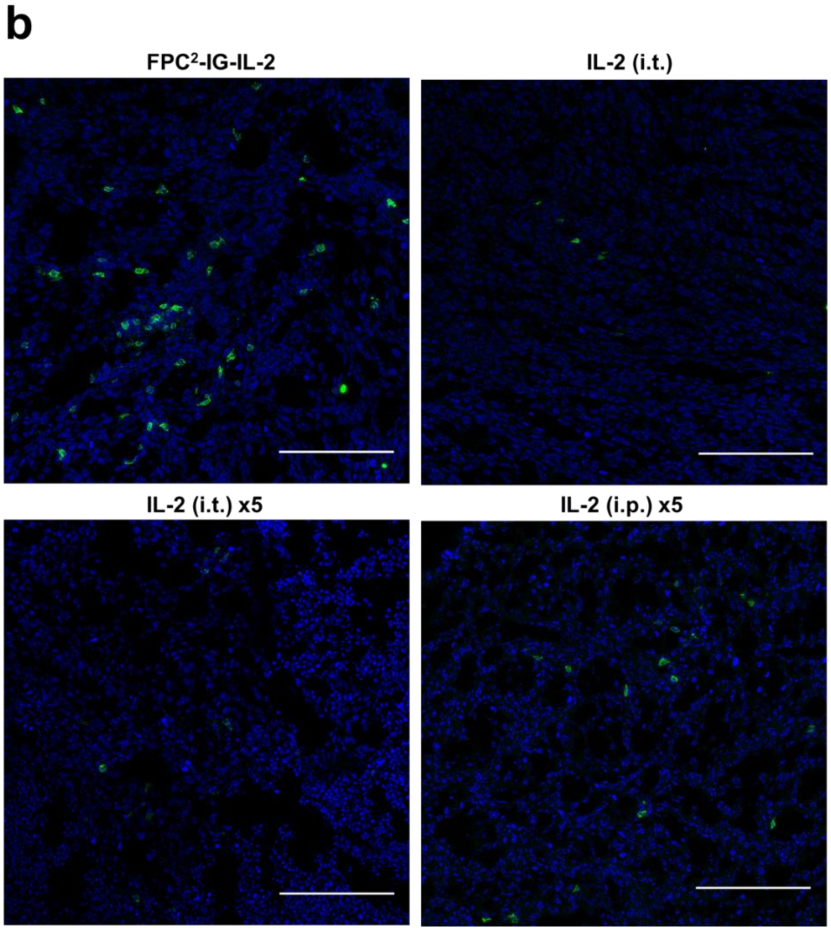

Supplementary Fig. 5. Effects of FPC^2 on immune cell activation. (a-c) Human PBMCs were incubated with PBS, Fucoidan and FPC^2 for 36 hrs. (a) Percent viability of human PBMCs measured by Zombie viability dye staining. (b) Frequencies of the indicated immune cell subsets in human PBMCs that were measured by FACS. (c) Frequencies of CD69 expression in the indicated immune cell subsets that were measured by FACS. (d) ELISA of IL-2, IFN-γ and TNF-α production by human PBMC cultured for 48 h in the presence of PBS, Fucoidan (20 μg/mL), FPC^2 (20 μg/mL). Concanavalin A (Con A; 5 μg/mL) was used as a positive control. Each dot represents an individual human sample. Data were compiled from three independent experiments. Statistical significance was determined by one-way ANOVA with Holm–Sidak multiple comparisons. ****p<0.0001, ns: not significant.

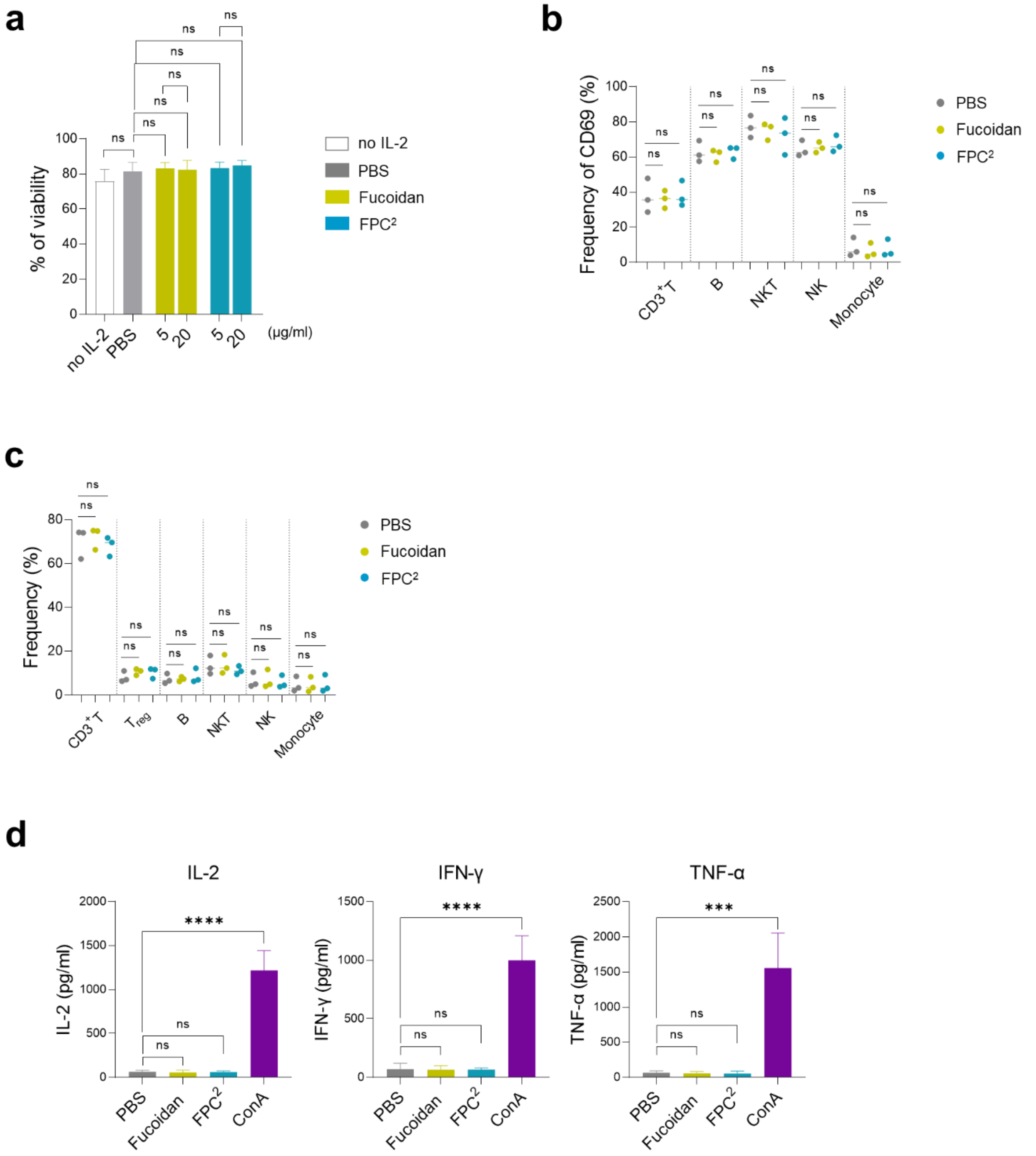
a
% of viability
no IL-2
PBS
Fucoidan
FPC²
ns
5
20
(μg/ml)
b
Frequency of CD69 (%)
CD3⁺T
B
NKT
NK
Monocyte
c
Frequency (%)
Treg
d
IL-2
IFN-γ
TNF-α
IL-2 (pg/ml)
IFN-γ (pg/ml)
TNF-α (pg/ml)
ConA
****
***

Supplementary Fig. 6. The Cancer Genome Atlas (TCGA) database analysis of IL-2 expression and T cell infiltration. (a) Kaplan–Meier curves for overall survival (OS) analysis according to the expressions of IL-2 in individuals with lung adenocarcinoma (left panel) and skin cutaneous melanoma (right panel). (b) Scatter plots showing the correlation between IL-2 expression and immune cell infiltration ($CD8^+$ T and Treg cells) in lung adenocarcinoma.

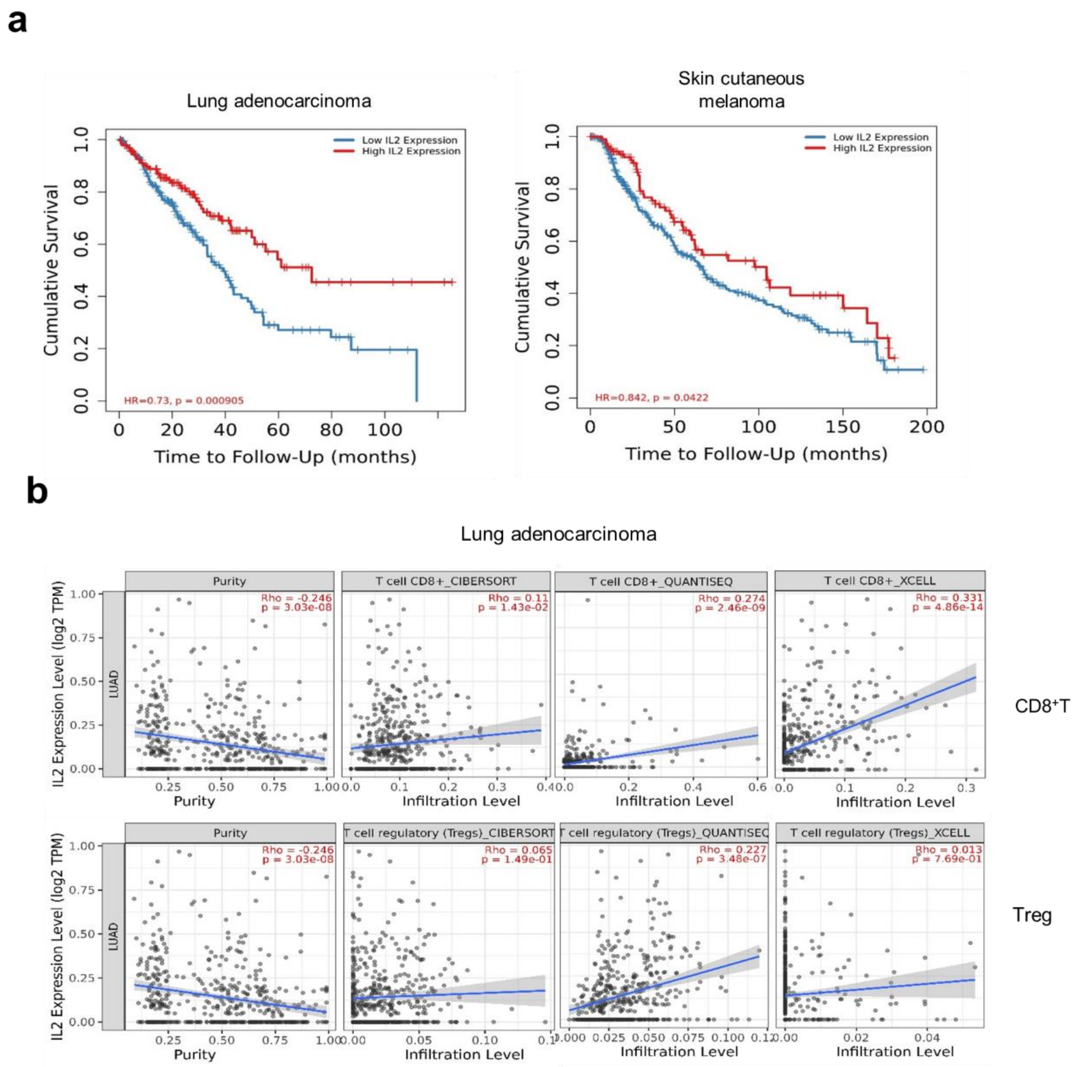

Supplementary Fig. 7. Gating strategy for identifying tumor infiltrating immune cell subsets by flow cytometry. FACS plots showing the indicated marker expression in single cell suspensions isolated from CT26 tumors.

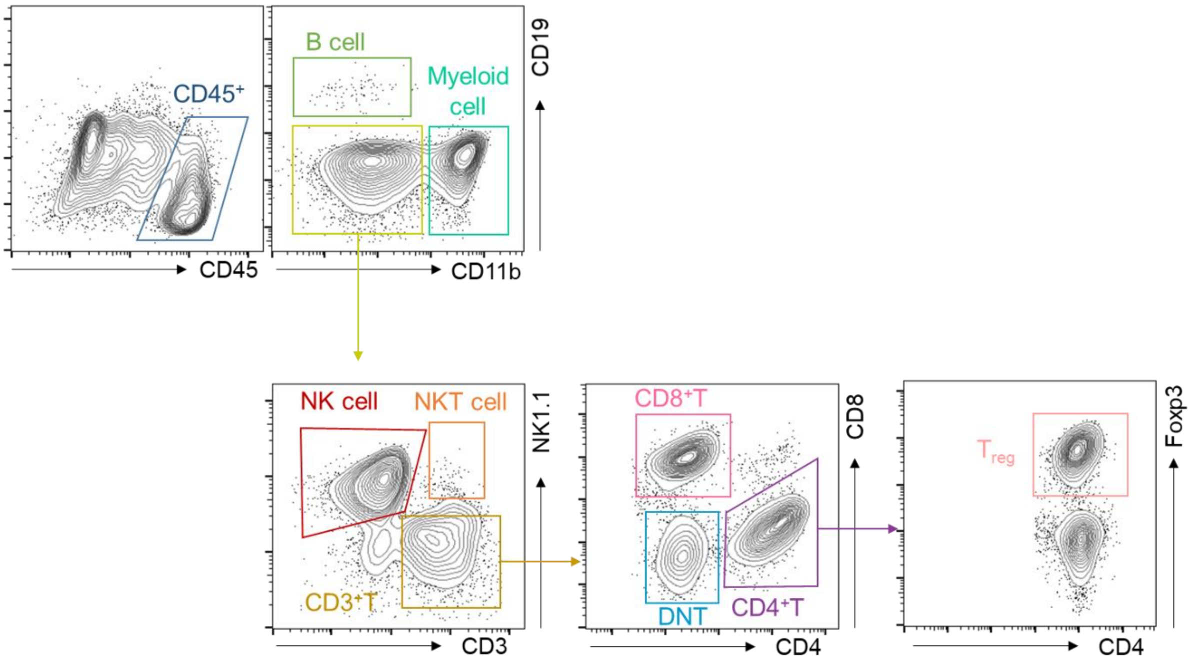

Supplementary Fig. 8. Relative binding ability of different compounds to IL-2. Different sulfated polysaccharide solutions (chondroitin sulfate A, fucoidan, and heparin) and fucoidan /PLL complex coacervates (FPC^2) were coated to each well of an ELISA plate, followed by incubation with different concentrations of IL-2 solution (1.25, 2.5, and 5 µg/mL). The amount of bound IL-2 was quantified using a sandwich ELISA method (n = 4). Relative IL-2 binding abilities were normalized to that of the sample with the highest value at each concentration of IL-2. All values shown are means ± SD. Statistical significance was designated as $p^{*} < 0.05$, $p^{**} < 0.01$, ns: not significant.

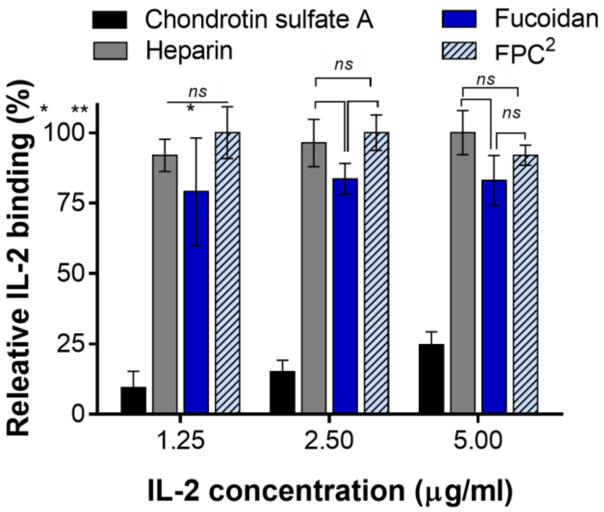

Supplementary Fig. 9. Precipitation after incubation of heparin and PLL. (a) Incubation of anionic heparin and cationic PLL caused precipitation rather that coacervation at physiological condition. (b) An excess addition of salt (more than 1 M NaCl) could induce complex coacervation by weakening strong electrostatic interactions. Scale bar = 100 μm.

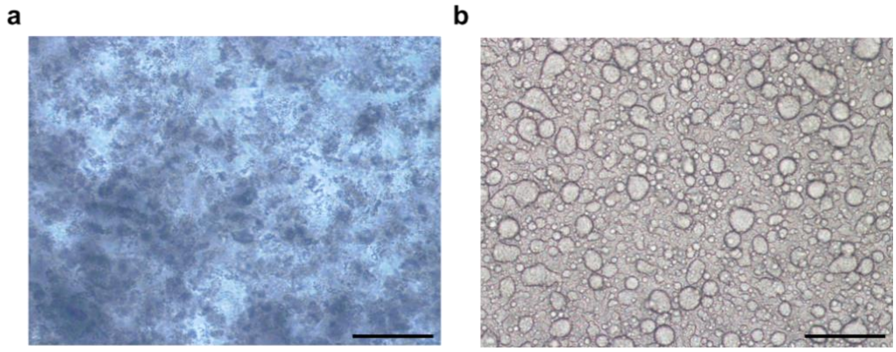

Materials and methods

Underwater stability of bulk FPC^2

To assess the underwater stability of FPC^2, the letter 'KIST' was written in a petri-dish containing PBS using bulk FPC^2 through a 22G syringe. The written bulk FPC^2 was incubated in PBS at RT and monitored for 2 months. Bulk FPC^2 was prepared by centrifugation of FPC^2 suspension at 12,000 g rpm and 4 °C for 10 min.

Incubation of heparin and PLL

Heparin from *porcine intestinal mucosa* (Sigma-Aldrich) was incubated with PLL dissolved in PBS or PBS containing 1 M NaCl (Sigma-Aldirch) at various weight ratios of heparin and PLL (0:10-10:0). The mixtures were observed by an optical microscope. Stock solutions of 6.25 mg/mL of each polyelectrolyte were prepared, and the total polyelectrolyte concentration was fixed at 6.25 mg/mL.

IL-2 binding affinity test

200 µL of fucoidan, chondroitin sulfate A from *bovine cartilage* (Sigma-Aldrich), heparin dissolved in PBS and FPC^2 (the weight ratio of fucoidan and PLL = 7:3) were incubated in a high-binding 96-well plate (Thermo Fisher) at a final concentration of 25 µg/mL overnight at RT. The coated-wells were then washed three times with PBS, and then incubated in 1% BSA (Sigma-Aldrich) in PBS for 1 h at 37 °C to inhibit non-specific binding. After washing with PBS, 200 µL of human interleukin-2 (IL-2, Peprotech) dissolved in PBS was added into each well at 0, 1.25, 2.5, and 5.0 µg/mL and incubated at room temperature for 2 h at 37 °C. Unbound IL-2 was removed by washing three times with PBS, and 100 µL of biotinylated anti IL-2 solution (Peprotech) was added, followed by a further incubation for 90 min at 37°C. After further washing of the wells three times with PBS, 100 µL of avidin-peroxidase (HRP) solution (Peprotech) was added and further incubated for 30 min at 37°C. After again washing three times with PBS, 100 µL of 2,2'-Azinobis [3-ethylbenzothiazoline-6-sulfonic acid]-diammonium salt (ABTS) liquid substrate solution (Peprotech) was added followed by a further incubation for 20 min at RT for color development. The IL-2 binding ability of each sample was calculated by measuring the absorbance at 450 nm, with the correction wavelength set at 650 nm. Relative IL-2 binding abilities were normalized to that of the sample with the highest value at each concentration of IL-2. Four independent samples were averaged to obtain each measurement.

The Cancer Genome Atlas (TCGA) analysis

Overall survival (OS) analysis and immune cell infiltration analysis were conducted on the TIMER2.0 web portal (http://timer.cistrome.org/). For OS analysis, the TCGA samples were divided into high and low cohorts according to the 25 % cut-off expression of the gene. Hazard ratios (HR) and log-rank *p* values were both calculated. TIMER, CIBERSORT, xCell and quanTIseq algorithms were used for immune cell infiltration analysis [27].
